# Ensemble Estimation of Information Divergence †

**DOI:** 10.3390/e20080560

**Published:** 2018-07-27

**Authors:** Kevin R. Moon, Kumar Sricharan, Kristjan Greenewald, Alfred O. Hero

**Affiliations:** 1 Genetics Department and Applied Math Program, Yale University, New Haven, CT 06520, USA; 2 Intuit Inc., Mountain View, CA 94043, USA; 3 IBM Research, Cambridge, MA 02142, USA; 4 Electrical Engineering and Computer Science Department, University of Michigan, Ann Arbor, MI 48109, USA

**Keywords:** divergence, differential entropy, nonparametric estimation, central limit theorem, convergence rates, bayes error rate

## Abstract

Recent work has focused on the problem of nonparametric estimation of information divergence functionals between two continuous random variables. Many existing approaches require either restrictive assumptions about the density support set or difficult calculations at the support set boundary which must be known a priori. The mean squared error (MSE) convergence rate of a leave-one-out kernel density plug-in divergence functional estimator for general bounded density support sets is derived where knowledge of the support boundary, and therefore, the boundary correction is not required. The theory of optimally weighted ensemble estimation is generalized to derive a divergence estimator that achieves the parametric rate when the densities are sufficiently smooth. Guidelines for the tuning parameter selection and the asymptotic distribution of this estimator are provided. Based on the theory, an empirical estimator of Rényi-α divergence is proposed that greatly outperforms the standard kernel density plug-in estimator in terms of mean squared error, especially in high dimensions. The estimator is shown to be robust to the choice of tuning parameters. We show extensive simulation results that verify the theoretical results of our paper. Finally, we apply the proposed estimator to estimate the bounds on the Bayes error rate of a cell classification problem.

## 1. Introduction

Information divergences are integral functionals of two probability distributions and have many applications in the fields of information theory, statistics, signal processing, and machine learning. Some applications of divergences include estimating the decay rates of error probabilities [1], estimating bounds on the Bayes error [2,3,4,5,6,7,8] or the minimax error [9] for a classification problem, extending machine learning algorithms to distributional features [10,11,12,13], testing the hypothesis that two sets of samples come from the same probability distribution [14], clustering [15,16,17], feature selection and classification [18,19,20], blind source separation [21,22], image segmentation [23,24,25], and steganography [26]. For many more applications of divergence measures, see reference [27]. There are many information divergence families including Alpha- and Beta-divergences [28] as well as *f*-divergences [29,30]. In particular, the *f*-divergence family includes the well-known Kullback–Leibler (KL) divergence [31], the Rényi-α divergence integral [32], the Hellinger–Bhattacharyya distance [33,34], the Chernoff-α divergence [5], the total variation distance, and the Henze–Penrose divergence [6].

Despite the many applications of divergences between continuous random variables, there are no nonparametric estimators of these functionals that achieve the parametric mean squared error (MSE) convergence rate, are simple to implement, do not require knowledge of the boundary of the density support set, and apply to a large set of divergence functionals. In this paper, we present the first information divergence estimator that achieves all of the above. Specifically, we address the problem of estimating divergence functionals when only a finite population of independent and identically distributed (i.i.d.) samples is available from the two d-dimensional distributions that are unknown, nonparametric, and smooth. Our contributions are as follows:

1. We propose the first information divergence estimator, referred to as EnDive, that is based on ensemble methods. The ensemble estimator takes a weighted average of an ensemble of weak kernel density plug-in estimators of divergence where the weights are chosen to improve the MSE convergence rate. This ensemble construction makes it very easy to implement EnDive.
2. We prove that the proposed ensemble divergence estimator achieves the optimal parametric MSE rate of $O\left(\frac{1}{N}\right)$, where *N* is the sample size when the densities are sufficiently smooth. In particular, EnDive achieves these rates without explicitly performing boundary correction which is required for most other estimators. Furthermore, we show that the convergence rates are uniform.
3. We prove that EnDive obeys a central limit theorem and thus, can be used to perform inference tasks on the divergence such as testing that two populations have identical distributions or constructing confidence intervals.

### 1.1. Related Work

Much work has focused on the problem of estimating the entropy and the information divergence of discrete random variables [1,29,35,36,37,38,39,40,41,42,43]. However, the estimation problem for discrete random variables differs significantly from the continuous case and thus employs different tools for both estimation and analysis.

One approach to estimating the differential entropy and information divergence of continuous random variables is to assume a parametric model for the underlying probability distributions [44,45,46]. However, these methods perform poorly when the parametric model does not fit the data well. Unfortunately, the structure of the underlying data distribution is unknown for many applications, and thus the chance for model misspecification is high. Thus, in many of these applications, parametric methods are insufficient, and nonparametric estimators must be used.

While several nonparametric estimators of divergence functionals between continuous random variables have been previously defined, the convergence rates are known for only a few of them. Furthermore, the asymptotic distributions of these estimators are unknown for nearly all of them. For example, Póczos and Schneider [10] established a weak consistency for a bias-corrected *k*-nearest neighbor (nn) estimator for Rényi-α and other divergences of a similar form where *k* was fixed. Li et al. [47] examined *k*-nn estimators of entropy and the KL divergence using hyperspherical data. Wang et al. [48] provided a *k*-nn based estimator for KL divergence. Plug-in histogram estimators of mutual information and divergence have been proven to be consistent [49,50,51,52]. Hero et al. [53] provided a consistent estimator for Rényi-α divergence when one of the densities is known. However none of these works studied the convergence rates or the asymptotic distribution of their estimators.

There has been recent interest in deriving convergence rates for divergence estimators for continuous data [54,55,56,57,58,59,60]. The rates are typically derived in terms of a smoothness condition on the densities, such as the Hölder condition [61]:

**Definition** **1** (Hölder Class)**.** *Let $X\subset R^{d}$ be a compact space. For $r=(r_{1},\ldots ,r_{d}),$$r_{i}\in N,$ define $\left|r\right|=\sum_{i=1}^{d}r_{i}$ and $D^{r}=\frac{\partial^{\left|r\right|}}{\partial x_{1}^{r_{1}}\ldots \partial x_{d}^{r_{d}}}$. The Hölder class $\Sigma (s,K_{H})$ of functions on $L_{2}\left(X\right)$ consists of the functions (f) that satisfy*

$$\left|D^{r}f\left(x\right)-D^{r}f\left(y\right)\right|\leq K_{H}{∥x-y∥}^{\min(s-|r|,1)},$$

*for all x,y∈X and for all r s.t. $\left|r\right|\leq \left\lfloor s\right\rfloor$.*

From Definition 1, it is clear that if a function (*f*) belongs to $\Sigma (s,K_{H})$, then *f* is continuously differentiable up to order $\left\lfloor s\right\rfloor$. In this work, we show that EnDive achieves a parametric MSE convergence rate of O(1/N) when s≥d and $s>\frac{d}{2}$, depending on the specific form of the divergence function.

Nguyen et al. [56] proposed an *f*-divergence estimator that estimates the likelihood ratio of the two densities by solving a convex optimization problem and then plugging it into the divergence formulas. The authors proved that the minimax MSE convergence rate is parametric when the likelihood ratio is a member of the bounded Hölder class $\Sigma (s,K_{H})$ with s≥d/2. However, this estimator is restricted to true *f*-divergences and may not apply to the broader class of divergence functionals that we consider here (as an example, the $L_{2}^{2}$ divergence is not an *f*-divergence). Additionally, solving the convex problem of [56] has similar computational complexity to that of training a support vector machine (SVM) (between $O(N^{2})$ and $O(N^{3})$), which can be demanding when *N* is large. In contrast, the EnDive estimator that we propose requires only the construction of simple density plug-in estimates and the solution of an offline convex optimization problem. Therefore, the most computationally demanding step in the EnDive estimator is the calculation of the density estimates, which has a computational complexity no greater than $O(N^{2})$.

Singh and Póczos [58,59] provided an estimator for Rényi-α divergences as well as general density functionals that use a “mirror image” kernel density estimator. They proved that these estimators obtain an MSE convergence rate of $O\left(\frac{1}{N}\right)$ when s≥d for each of the densities. However their approach requires several computations at each boundary of the support of the densities which is difficult to implement as *d* gets large. Also, this computation requires knowledge of the support (specifically, the boundaries) of the densities which is unknown in most practical settings. In contrast, while our assumptions require the density support sets to be bounded and the boundaries to be smooth, knowledge of the support is not required to implement EnDive.

The “linear” and “quadratic” estimators presented by Krishnamurthy et al. [57] estimate divergence functionals that include the form $\int f_{1}^{\alpha }\left(x\right)f_{2}^{\beta }\left(x\right)d\mu \left(x\right)$ for given α and β where $f_{1}$ and $f_{2}$ are probability densities. These estimators achieve the parametric rate when s≥d/2 and s≥d/4 for the linear and quadratic estimators, respectively. However, the latter estimator is computationally infeasible for most functionals, and the former requires numerical integration for some divergence functionals which can be computationally difficult. Additionally, while a suitable α-β indexed sequence of divergence functionals of this form can be constructed that converge to the KL divergence, this does not guarantee convergence of the corresponding sequence of divergence estimators, as shown in reference [57]. In contrast, EnDive can be used to estimate the KL divergence directly. Other important *f*-divergence functionals are also excluded from this form including some that bound the Bayes error [2,4,6]. In contrast, our method applies to a large class of divergence functionals and avoids numerical integration.

Finally, Kandasamy et al. [60] proposed influence function-based estimators of distributional functionals including divergences that achieve the parametric rate when s≥d/2. While this method can be applied to general functionals, the estimator requires numerical integration for some functionals. Additionally, the estimators in both Kandasamy et al. [60] and Krishnamurthy et al. [57] require an optimal kernel density estimator. This is difficult to construct when the density support is bounded as it requires difficult computations at the density support set boundary and therefore, knowledge of the density support set. In contrast, Endive does not require knowledge of the support boundary.

In addition to the MSE convergence rates, the asymptotic distribution of divergence estimators is of interest. Asymptotic normality has been established for certain divergences between a specific density estimator and the true density [62,63,64]. This differs from the problem we consider where we assume that both densities are unknown. The asymptotic distributions of the estimators in references [56,57,58,59] are currently unknown. Thus, it is difficult to use these estimators for hypothesis testing which is crucial in many scientific applications. Kandasamy et al. [60] derived the asymptotic distribution of their data-splitting estimator but did not prove similar results for their leave-one-out estimator. We establish a central limit theorem for EnDive which greatly enhances its applicability in scientific settings.

Our ensemble divergence estimator reduces to an ensemble entropy estimator as a special case when data from only one distribution is considered and the other density is set to a uniform measure (see reference [28] for more on the relationship between entropy and information divergence). The resultant entropy estimator differs from the ensemble entropy estimator proposed by Sricharan et al. [65] in several important ways. First, the density support set must be known for the estimator in reference [65] to perform the explicit boundary correction. In contrast, the EnDive estimator does not require any boundary correction. To show this requires a significantly different approach to prove the bias and variance rates of the EnDive estimator. Furthermore, the EnDive results apply under more general assumptions for the densities and the kernel used in the weak estimators. Finally, the central limit theorem applies to the EnDive estimator which is currently unknown for the estimator in reference [65].

We also note that Berrett et al. [66] proposed a modification of the Kozachenko and Leonenko estimator of entropy [67] that takes a weighted ensemble estimation approach. While their results require stronger assumptions for the smoothness of the densities than ours do, they did obtain the asymptotic distribution of their weighted estimator and they also showed that the asymptotic variance of the estimator is not increased by taking a weighted average. This latter point is an important selling point of the ensemble framework—we can improve the asymptotic bias of an estimator without increasing the asymptotic variance.

### 1.2. Organization and Notation

The paper is organized as follows. We first derive the MSE convergence rates in Section 2 for a weak divergence estimator, which is a kernel density plug-in divergence estimator. We then generalize the theory of optimally weighted ensemble entropy estimation developed in reference [65] to obtain the ensemble divergence estimator EnDive from an ensemble of weak estimators in Section 3. A central limit theorem and uniform convergence rate for the ensemble estimator are also presented in Section 3. In Section 4, we provide guidelines for selecting the tuning parameters based on experiments and the theory derived in the previous sections. We then perform experiments in Section 4 that validate the theory and establish the robustness of the proposed estimators to the tuning parameters.

Bold face type is used for random variables and random vectors. The conditional expectation given a random variable Z is denoted as $E_{Z}$. The variance of a random variable is denoted as V, and the bias of an estimator is denoted as B.

## 2. The Divergence Functional Weak Estimator

This paper focuses on estimating functionals of the form

(1) $$G\left(f_{1},f_{2}\right)=\int g\left(f_{1}\left(x\right),f_{2}\left(x\right)\right)f_{2}\left(x\right)dx,,$$

where g(x,y) is a smooth functional, and $f_{1}$ and $f_{2}$ are smooth *d*-dimensional probability densities. If $g\left(f_{1}\left(x\right),f_{2}\left(x\right)\right)=g\left(\frac{f_{1}\left(x\right)}{f_{2}\left(x\right)}\right),$ *g* is convex, and g(1)=0, then $G\left(f_{1},f_{2}\right)$ defines the family of *f*-divergences. Some common divergences that belong to this family include the KL divergence (g(t)=−lnt) and the total variation distance (g(t)=|t−1|). In this work, we consider a broader class of functionals than the *f*-divergences, since *g* is allowed to be very general.

To estimate $G\left(f_{1},f_{2}\right)$, we first define a weak plug-in estimator based on kernel density estimators (KDEs), that is, a simple estimator that converges slowly to the true value $G\left(f_{1},f_{2}\right)$ in terms of MSE. We then derive the bias and variance expressions for this weak estimator as a function of sample size and bandwidth. We then use the resulting bias and variance expressions to derive an ensemble estimator that takes a weighted average of weak estimators with different bandwidths and achieves superior MSE performance.

### 2.1. The Kernel Density Plug-in Estimator

We use a kernel density plug-in estimator of the divergence functional in (1) as the weak estimator. Assume that $N_{1}$ i.i.d. realizations $\left\{Y_{1},\ldots ,Y_{N_{1}}\right\}$ are available from $f_{1}$ and $N_{2}$ i.i.d. realizations $\left\{X_{1},\ldots ,X_{N_{2}}\right\}$ are available from $f_{2}$. Let $h_{i}>0$ be the kernel bandwidth for the density estimator of $f_{i}$. For simplicity of presentation, assume that $N_{1}=N_{2}=N$ and $h_{1}=h_{2}=h$. The results for the more general case of differing sample sizes and bandwidths are given in Appendix C. Let K(·) be a kernel function with ∫K(x)dx=1 and ${\left||K|\right|}_{\infty }<\infty$ where ${∥K∥}_{\infty }$ is the $\ell_{\infty }$ norm of the kernel (*K*). The KDEs for $f_{1}$ and $f_{2}$ are, respectively,

$$\begin{array}{ccc} {\tilde{f}}_{1,h}\left(X_{j}\right) & = & \frac{1}{Nh^{d}}\sum_{i=1}^{N}K\left(\frac{X_{j}-Y_{i}}{h}\right),\\ {\tilde{f}}_{2,h}\left(X_{j}\right) & = & \frac{1}{Mh^{d}}\sum_{\begin{array}{c} i=1\\ i\neq j \end{array}}^{N}K\left(\frac{X_{j}-X_{i}}{h}\right), \end{array}$$

where M=N−1. $G\left(f_{1},f_{2}\right)$ is then approximated as

(2) $${\tilde{G}}_{h}=\frac{1}{N}\sum_{i=1}^{N}g\left({\tilde{f}}_{1,h}\left(X_{i}\right),{\tilde{f}}_{2,h}\left(X_{i}\right)\right).$$

### 2.2. Convergence Rates

For many estimators, MSE convergence rates are typically provided in the form of upper (or sometimes lower) bounds on the bias and the variance. Therefore, only the slowest converging terms (as a function of the sample size (*N*)) are presented in these cases. However, to apply our generalized ensemble theory to obtain estimators that guarantee the parametric MSE rate, we required explicit expressions for the bias of the weak estimators in terms of the sample size (*N*) and the kernel bandwidth (*h*). Thus, an upper bound was insufficient for our work. Furthermore, to guarantee the parametric rate, we required explicit expressions of all bias terms that converge to zero slower than $O\left(1/\sqrt{N}\right)$.

To obtain bias expressions, we required multiple assumptions on the densities $f_{1}$ and $f_{2}$, the functional *g*, and the kernel *K*. Similar to reference [7,54,65], the principal assumptions we make were that (1) $f_{1}$, $f_{2},$ and *g* are smooth; (2) $f_{1}$ and $f_{2}$ have common bounded support sets S; and (3) $f_{1}$ and $f_{2}$ are strictly lower bounded on S. We also assume (4) that the density support set is smooth with respect to the kernel (K(u)). The full technical assumptions and a discussion of them are contained in Appendix A. Given these assumptions, we have the following result on the bias of ${\tilde{G}}_{h}$:

**Theorem** **1.** *For a general g, the bias of the plug-in estimator ${\tilde{G}}_{h}$ is given by*

(3) $$B\left[{\tilde{G}}_{h}\right]=\sum_{j=1}^{\left\lfloor s\right\rfloor }c_{10,j}h^{j}+c_{11}\frac{1}{Nh^{d}}+O\left(h^{s}+\frac{1}{Nh^{d}}\right).$$

To apply our generalized ensemble theory to the KDE plug-in estimator (${\tilde{G}}_{h}$), we required only an upper bound on its variance. The following variance result required much less strict assumptions than the bias results in Theorem 1:

**Theorem** **2.** *Assume that the functional g in (1) is Lipschitz continuous in both of its arguments with the Lipschitz constant ($C_{g}$). Then, the variance of the plug-in estimator (${\tilde{G}}_{h}$) is bounded by*

$$V\left[{\tilde{G}}_{h}\right]\leq C_{g}^{2}{\left||K|\right|}_{\infty }^{2}\frac{11}{N}.$$

From Theorems 1 and 2, we observe that h→0 and $Nh^{d}\to \infty$ are required for ${\tilde{G}}_{h}$ to be unbiased, while the variance of the plug-in estimator depends primarily on the sample size (*N*). Note that the constants depend on the densities $f_{1}$ and $f_{2}$ and their derivatives which are often unknown.

### 2.3. Optimal MSE Rate

From Theorem 1, the dominating terms in the bias are observed to be $\Theta \left(h\right)$ and $\Theta \left(\frac{1}{Nh^{d}}\right)$. If no bias correction is performed, the optimal choice of *h* that minimizes MSE is

$$h^{*}=\Theta \left(N^{\frac{-1}{d+1}}\right).$$

This results in a dominant bias term of order $\Theta \left(N^{\frac{-1}{d+1}}\right)$. Note that this differs from the standard result for the optimal KDE bandwidth for minimum MSE density estimation which is $\Theta \left(N^{-1/\left(d+4\right)}\right)$ for a symmetric uniform kernel when the boundary bias is ignored [68].

**Figure 1:**
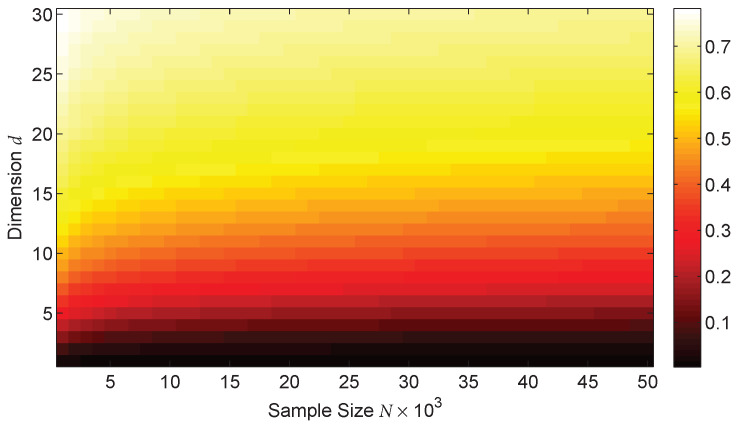
Heat map showing the predicted bias of the divergence functional plug-in estimator ${\tilde{G}}_{h}$ based on Theorem 1 as a function of the dimensions (*d*) and sample size (*N*) when $h=N^{\frac{-1}{d+1}}$. Note that the phase transition in the bias as the dimensions (*d*) increase for a fixed sample size (*N*); the bias remains small only for relatively small values of d. The proposed weighted ensemble estimator EnDive eliminates this phase transition when the densities and the function *g* are sufficiently smooth.

Figure 1 shows a heatmap showing the leading bias term $O\left(h\right)$ as a function of *d* and *N* when $h=N^{\frac{-1}{d+1}}$. The heatmap indicates that the bias of the plug-in estimator in (2) is small only for relatively small values of *d*. This is consistent with the empirical results in reference [69] which examined the MSE of multiple plug-in KDE and *k*-nn estimators. In the next section, we propose an ensemble estimator that achieves a superior convergence rate regardless of the dimensions (*d*) as long as the density is sufficiently smooth.

### 2.4. Proof Sketches of Theorems 1 and 2

To prove the bias expressions in Theorem 1, the bias is first decomposed into two parts by adding and subtracting $g\left(E_{Z}{\tilde{f}}_{1,h}\left(Z\right),E_{Z}{\tilde{f}}_{2,h}\left(Z\right)\right)$ within the expectation creating a “bias” term and a “variance” term. Applying a Taylor series expansion on the bias and variance terms results in expressions that depend on powers of $B_{Z}\left[{\tilde{f}}_{i,h}\left(Z\right)\right]:=E_{Z}{\tilde{f}}_{i,h}\left(Z\right)-f_{i}\left(Z\right)$ and ${\tilde{e}}_{i,h}\left(Z\right):={\tilde{f}}_{i,h}\left(Z\right)-E_{Z}{\tilde{f}}_{i,h}\left(Z\right)$, respectively. Within the interior of the support, moment bounds can be derived from properties of the KDEs and a Taylor series expansion of the densities. Near the boundary of the support, the smoothness assumption on the boundary A.5 is required to obtain an expression of the bias in terms of the KDE bandwidth (*h*) and the sample size (*N*). The full proof of Theorem 1 is given in Appendix E.

The proof of the variance result takes a different approach. The proof uses the Efron–Stein inequality [70] which bounds the variance by analyzing the expected squared difference between the plug-in estimator when one sample is allowed to differ. This approach provides a bound on the variance under much less strict assumptions on the densities and the functional *g* than is required for Theorem 1. The full proof of Theorem 2 is given in Appendix F.

## 3. Weighted Ensemble Estimation

From Theorem 1 and Figure 1, we can observe that the bias of the MSE-optimal plug-in estimator ${\tilde{G}}_{h}$ decreases very slowly as a function of the sample size (*N*) when the data dimensions (*d*) are not small, resulting in a large MSE. However, by applying the theory of optimally weighted ensemble estimation, we can obtain an estimator with improved performance by taking a weighted sum of an ensemble of weak estimators where the weights are chosen to significantly reduce the bias.

The ensemble of weak estimators is formed by choosing different values of the bandwidth parameter *h* as follows. Set $L=\left\{l_{1},\ldots ,l_{L}\right\}$ to be real positive numbers that index $h(l_{i})$. Thus, the parameter *l* indexes over different neighborhood sizes for the KDEs. Define the weight $w:=\left\{w\left(l_{1}\right),\ldots ,w\left(l_{L}\right)\right\}$ and ${\tilde{G}}_{w}:=\sum_{l\in L}w\left(l\right){\tilde{G}}_{h(l)}.$ That is, for each estimator ${\tilde{G}}_{h(l)}$ there is a corresponding weight value (w(l)). The key to reducing the MSE is to choose the weight vector (*w*) to reduce the lower order terms in the bias while minimizing the impact of the weighted average on the variance.

### 3.1. Finding the Optimal Weight

The theory of optimally weighted ensemble estimation is a general theory that is applicable to any estimation problem as long as the bias and variance of the estimator can be expressed in a specific way. An early version of this theory was presented in reference [65]. We now generalize this theory so that it can be applied to a wider variety of estimation problems. Let *N* be the number of available samples and let $L=\left\{l_{1},\ldots ,l_{L}\right\}$ be a set of index values. Given an indexed ensemble of estimators ${\left\{{\hat{E}}_{l}\right\}}_{l\in L}$ of some parameter (*E*), the weighted ensemble estimator with weights $w=\left\{w\left(l_{1}\right),\ldots ,w\left(l_{L}\right)\right\}$ satisfying $\sum_{l\in L}w\left(l\right)=1$ is defined as

$${\hat{E}}_{w}=\sum_{l\in L}w\left(l\right){\hat{E}}_{l}.$$

${\hat{E}}_{w}$ is asymptotically unbiased as long as the estimators ${\left\{{\hat{E}}_{l}\right\}}_{l\in L}$ are asymptotically unbiased. Consider the following conditions on ${\left\{{\hat{E}}_{l}\right\}}_{l\in L}$:

- C.1 The bias is expressible as
  $$B\left[{\hat{E}}_{l}\right]=\sum_{i\in J}c_{i}\psi_{i}\left(l\right)\phi_{i,d}\left(N\right)+O\left(\frac{1}{\sqrt{N}}\right),$$
  where $c_{i}$ are constants that depend on the underlying density and are independent of *N* and *l*, $J=\left\{i_{1},\ldots ,i_{I}\right\}$ is a finite index set with I<L, and $\psi_{i}\left(l\right)$ are basis functions depending only on parameter *l* and not on the sample size (*N*).
- C.2 The variance is expressible as
  $$V\left[{\hat{E}}_{l}\right]=c_{v}\left(\frac{1}{N}\right)+o\left(\frac{1}{N}\right).$$

**Theorem** **3.** *Assume conditions C.1 and C.2 hold for an ensemble of estimators ${\left\{{\hat{E}}_{l}\right\}}_{l\in L}$. Then, there exists a weight vector ($w_{0}$) such that the MSE of the weighted ensemble estimator attains the parametric rate of convergence:*

$$E\left[{\left({\hat{E}}_{w_{0}}-E\right)}^{2}\right]=O\left(\frac{1}{N}\right).$$

*The weight vector ($w_{0}$) is the solution to the following convex optimization problem:*

(4) $$\begin{array}{cc} \min_{w} & {\left||w|\right|}_{2}\\ subject\;to & \sum_{l\in L}w\left(l\right)=1,\\ & \gamma_{w}\left(i\right)=\sum_{l\in L}w\left(l\right)\psi_{i}\left(l\right)=0,\;i\in J. \end{array}$$

**Proof.**  From condition C.1, we can write the bias of the weighted estimator as

$$B\left[{\hat{E}}_{w}\right]=\sum_{i\in J}c_{i}\gamma_{w}\left(i\right)\phi_{i,d}\left(N\right)+O\left(\frac{\sqrt{L}{\left||w|\right|}_{2}}{\sqrt{N}}\right).$$

The variance of the weighted estimator is bounded as

(5) $$V\left[{\hat{E}}_{w}\right]\leq \frac{{L||w||}_{2}^{2}}{N}.$$

The optimization problem in (4) zeroes out the lower-order bias terms and limits the $\ell_{2}$ norm of the weight vector (*w*) to prevent the variance from exploding. This results in an MSE rate of O(1/N) when the dimensions (*d*) are fixed and when *L* is fixed independently of the sample size (*N*). Furthermore, a solution to (4) is guaranteed to exist if L>I and the vectors $a_{i}=\left[\psi_{i}\left(l_{1}\right),\ldots ,\psi_{i}\left(l_{L}\right)\right]$ are linearly independent. This completes our sketch of the proof of Theorem 3. ☐

### 3.2. The EnDive Estimator

The parametric rate of $O\left(1/N\right)$ in MSE convergence can be achieved without requiring $\gamma_{w}\left(i\right)=0,\;i\in J$. This can be accomplished by solving the following convex optimization problem in place of the optimization problem in Theorem 3:

(6) $$\begin{array}{cc} \min_{w} & \epsilon \\ subject\;to & \sum_{l\in L}w\left(l\right)=1,\\ & \left|\gamma_{w}\left(i\right)N^{\frac{1}{2}}\phi_{i,d}\left(N\right)\right|\leq \epsilon ,\;\;i\in J,\\ & {∥w∥}_{2}^{2}\leq \eta \epsilon , \end{array}$$

where the parameter η is chosen to achieve a trade-off between bias and variance. Instead of forcing $\gamma_{w}\left(i\right)=0$, the relaxed optimization problem uses the weights to decrease the bias terms at a rate of $O\left(1/\sqrt{N}\right)$, yielding an MSE convergence rate of O(1/N). In fact, it was shown in reference [71] that the optimization problem in (6) guarantees the parametric MSE rate as long as the conditions of Theorem 3 are satisfied and a solution to the optimization problem in (4) exists (the conditions for this existence are given in the proof of Theorem 3).

We now construct a divergence ensemble estimator from an ensemble of plug-in KDE divergence estimators. Consider first the bias result in (3) where *g* is general, and assume that s≥d. In this case, the bias contains a $O\left(\frac{1}{h^{d}N}\right)$ term. To guarantee the parametric MSE rate, any remaining lower-order bias terms in the ensemble estimator must be no slower than $O\left(1/\sqrt{N}\right)$. Let $h\left(l\right)=lN^{-1/(2d)}$ where l∈L. Then $O\left(\frac{1}{h{\left(l\right)}^{d}N}\right)=O\left(\frac{1}{l^{d}\sqrt{N}}\right)$. We therefore obtain an ensemble of plug-in estimators ${\left\{{\tilde{G}}_{h(l)}\right\}}_{l\in L}$ and a weighted ensemble estimator ${\tilde{G}}_{w}=\sum_{l\in L}w\left(l\right){\tilde{G}}_{h(l)}$. The bias of each estimator in the ensemble satisfies the condition C.1 with $\psi_{i}\left(l\right)=l^{i}$ and $\phi_{i,d}\left(N\right)=N^{-i/(2d)}$ for i=1,…,d. To obtain a uniform bound on the bias with respect to *w* and L, we also include the function $\psi_{d+1}\left(l\right)=l^{-d}$ with corresponding $\phi_{d+1,d}\left(N\right)=N^{-1/2}$. The variance also satisfies the condition C.2. The optimal weight ($w_{0}$) is found by using (6) to obtain an optimally weighted plug-in divergence functional estimator ${\tilde{G}}_{w_{0}}$ with an MSE convergence rate of $O\left(\frac{1}{N}\right)$ as long as s≥d and L≥d. Otherwise, if s<d, we can only guarantee the MSE rate up to $O\left(\frac{1}{N^{s/d}}\right)$. We refer to this estimator as the Ensemble Divergence (EnDive) estimator and denote it as ${\tilde{G}}_{\mathrm{EnDive}}$.

We note that for some functionals (*g*) (including the KL divergence and the Renyi-α divergence integral), we can modify the EnDive estimator to obtain the parametric rate under the less strict assumption that s>d/2. For details on this approach, see Appendix B.

### 3.3. Central Limit Theorem

The following theorem shows that an appropriately normalized ensemble estimator ${\tilde{G}}_{w}$ converges in distribution to a normal random variable under rather general conditions. Thus, the same result applies to the EnDive estimator ${\tilde{G}}_{\mathrm{EnDive}}$. This enables us to perform hypothesis testing on the divergence functional which is very useful in many scientific applications. The proof is based on the Efron–Stein inequality and an application of Slutsky’s Theorem (Appendix G).

**Theorem** **4.** *Assume that the functional g is Lipschitz in both arguments with the Lipschitz constant $C_{g}$. Further assume that h(l)=o(1), N→∞, and $Nh{\left(l\right)}^{d}\to \infty$ for each l∈L. Then, for a fixed L, the asymptotic distribution of the weighted ensemble estimator ${\tilde{G}}_{w}$ is*

$$Pr\left(\left({\tilde{G}}_{w}-E\left[{\tilde{G}}_{w}\right]\right)/\sqrt{V\left[{\tilde{G}}_{w}\right]}\leq t\right)\to Pr\left(S\leq t\right),$$

*where S is a standard normal random variable.*

### 3.4. Uniform Convergence Rates

Here, we show that the optimally weighted ensemble estimators achieve the parametric MSE convergence rate uniformly. Denote the subset of $\Sigma \left(s,K_{H}\right)$ with densities bounded between $\epsilon_{0}$ and $\epsilon_{\infty }$ as $\Sigma \left(s,K_{H},\epsilon_{0},\epsilon_{\infty }\right)$.

**Theorem** **5.** *Let ${\tilde{G}}_{\mathrm{EnDive}}$ be the EnDive estimator of the functional*

$$G\left(p,q\right)=\int g\left(p(x),q(x)\right)q\left(x\right)dx,$$

*where p and q are d-dimensional probability densities. Additionally, let r=d and assume that s>r. Then,*

(7) $$\underset{p,q\in \Sigma \left(s,K_{H},\epsilon_{0},\epsilon_{\infty }\right)}{\sup}E\left[{\left({\tilde{G}}_{w_{0}}-G\left(p,q\right)\right)}^{2}\right]\leq \frac{C}{N},$$

*where C is a constant.*

The proof decomposes the MSE into the variance plus the square of the bias. The variance is bounded easily by using Theorem 2. To bound the bias, we show that the constants in the bias terms are continuous with respect to the densities *p* and *q* under an appropriate norm. We then show that $\Sigma \left(s,K_{H},\epsilon_{0},\epsilon_{\infty }\right)$ is compact with respect to this norm and then apply an extreme value theorem. Details are given in Appendix H.

## 4. Experimental Results

In this section, we discuss the choice of tuning parameters and validate the EnDive estimator’s convergence rates and the central limit theorem. We then use the EnDive estimator to estimate bounds on the Bayes error for a single-cell bone marrow data classification problem.

### 4.1. Tuning Parameter Selection

The optimization problem in (6) has parameters η, *L*, and L. By applying (6), and the resulting MSE of the ensemble estimator is

(8) $$O\left(\epsilon^{2}/N\right)+O\left(L\eta^{2}\epsilon^{2}/N\right),$$

where each term in the sum comes from the bias and variance, respectively. From this expression and (6), we see that the parameter η provides a tradeoff between bias and variance. Increasing η enables the norm of the weight vector to be larger. This means the feasible region for the variable *w* increases in size as η increases which can result in decreased bias. However, as η contributes to the variance term, increasing η may result in increased variance.

If all of the constants in (3) and an exact expression for the variance of the ensemble estimator were known, then η could be chosen to optimize this tradeoff in bias and variance and thus minimize the MSE. Since these constants are unknown, we can only choose η based on the asymptotic results. From (8), this would suggest setting $\eta =1/\sqrt{L}$. In practice, we find that for finite sample sizes, the variance in the ensemble estimator is less than the upper bound of $L\eta^{2}\epsilon^{2}/N$. Thus, setting $\eta =1/\sqrt{L}$ is unnecessarily restrictive. We find that, in practice, setting η=1 works well.

Upon first glance, it appears that for fixed *L*, the set L that parameterizes the kernel widths can, in theory, be chosen by minimizing ϵ in (6) over L in addition to *w*. However, adding this constraint results in a non-convex optimization problem since *w* does not lie in the non-negative orthant. A parameter search over possible values for L is another possibility. However, this may not be practical as ϵ generally decreases as the size and spread of L increases. In addition, for finite sample sizes, decreasing ϵ does not always directly correspond to a decrease in MSE, as very high or very low values of h(l) can lead to inaccurate density estimates, resulting in a larger MSE.

**Figure 2:**
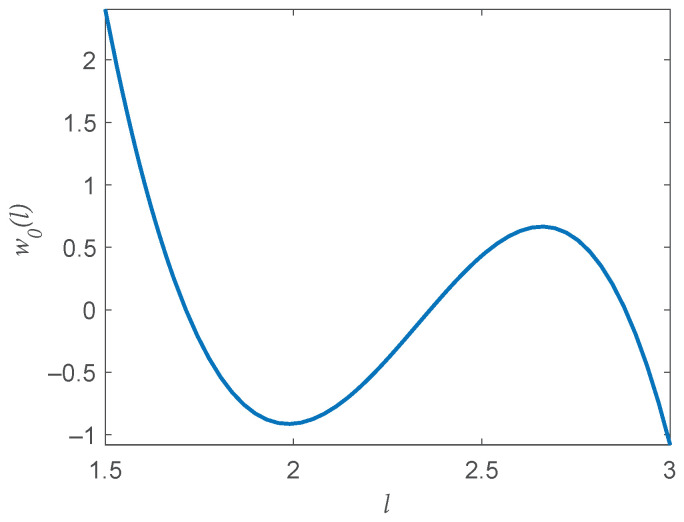
The optimal weights from (6) when d=4, N=3100, L=50, and *l* are uniformly spaced between 1.5 and 3. The lowest values of *l* are given the highest weight. Thus, the minimum value of bandwidth parameters L should be sufficiently large to render an adequate estimate.

Given these limitations, we provide the following recommendations for L. Denote the value of the minimum value of *l* such that ${\tilde{f}}_{i,h(l_{min})}\left(X_{j}\right)>0$∀i=1,2 as $l_{min}$ and the diameter of the support S as *D*. To ensure the KDEs are bounded away from zero, we require that $\min\left(L\right)\geq l_{min}$. As demonstrated in Figure 2, the weights in $w_{0}$ are generally largest for the smallest values of L. This indicates that min(L) should also be sufficiently larger than $l_{min}$ to render an adequate density estimate. Similarly, max(L) should be sufficiently smaller than the diamter (*D*) as high bandwidth values can lead to high bias in the KDEs. Once these values are chosen, all other L values can then be chosen to be equally spaced between min(L) and max(L).

An efficient way to choose $l_{min}$ and $l_{max}$ is to select the integers $k_{min}$ and $k_{max}$ and compute the $k_{min}$ and $k_{max}$ nearest neighbor distances of all the data points. The bandwidths $h(l_{min})$ and $h(l_{max})$ can then be chosen to be the maximums of these corresponding distances. The parameters $l_{min}$ and $l_{max}$ can then be computed from the expression $h\left(l\right)=lN^{-\frac{1}{2d}}$. This choice ensures that a minimum of $k_{min}$ points are within the kernel bandwidth for the density estimates at all points and that a maximum of $k_{max}$ points are within the kernel bandwidth for the density estimates at one of the points.

Once min(L) and max(L) have been chosen, the similarity of bandwidth values h(l) and basis functions $\psi_{i,d}\left(l\right)$ increases as *L* increases, resulting in a negligible decrease in the bias. Hence, *L* should be chosen to be large enough for sufficient bias but small enough so that the bandwidth values h(l) are sufficiently distinct. In our experiments, we found 30≤L≤60 to be sufficient.

### 4.2. Convergence Rates Validation: Rényi-α Divergence

To validate our theoretical convergence rate results, we estimated the Rényi-α divergence integral between two truncated multivariate Gaussian distributions with varying dimension and sample sizes. The densities had means of ${\bar{\mu }}_{1}=0.7\times {\bar{1}}_{d}$, ${\bar{\mu }}_{2}=0.3\times {\bar{1}}_{d}$ and covariance matrices of $0.4\times I_{d}$, where ${\bar{1}}_{d}$ is a *d*-dimensional vector of ones, and $I_{d}$ is a d×d identity matrix. We restricted the Gaussians to the unit cube and used α=0.5.

**Figure 3:**
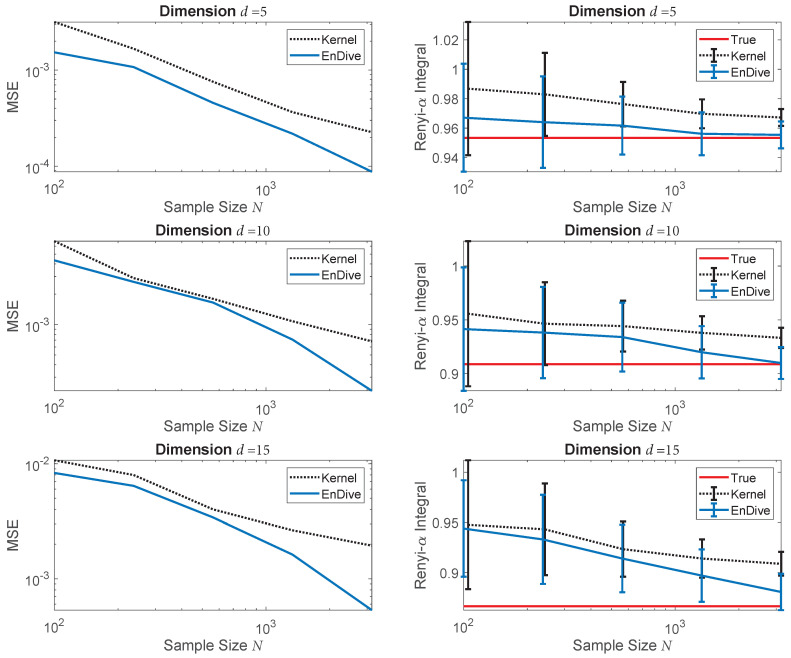
(**Left**) Log–log plot of MSE of the uniform kernel plug-in (“Kernel”) and the optimally weighted EnDive estimator for various dimensions and sample sizes. (**Right**) Plot of the true values being estimated compared to the average values of the same estimators with standard error bars. The proposed weighted ensemble estimator approaches the theoretical rate (see Table 1), performed better than the plug-in estimator in terms of MSE and was less biased.

The left plots in Figure 3 show the MSE (200 trials) of the standard plug-in estimator implemented with a uniform kernel and the proposed optimally weighted estimator EnDive for various dimensions and sample sizes. The parameter set L was selected based on a range of *k*-nearest neighbor distances. The bandwidth used for the standard plug-in estimator was selected by setting $h_{fixed}\left(l^{*}\right)=l^{*}N^{-\frac{1}{d+1}}$, where $l^{*}$ was chosen from L to minimize the MSE of the plug-in estimator. For all dimensions and sample sizes, EnDive outperformed the plug-in estimator in terms of MSE. EnDive was also less biased than the plug-in estimator and even had lower variance at smaller sample sizes (e.g., N=100). This reflects the strength of ensemble estimators—the weighted sum of a set of relatively poor estimators can result in a very good estimator. Note also that for the larger values of *N*, the ensemble estimator MSE rates approached the theoretical rate based on the estimated log–log slope given in Table 1.

**Figure 4:**
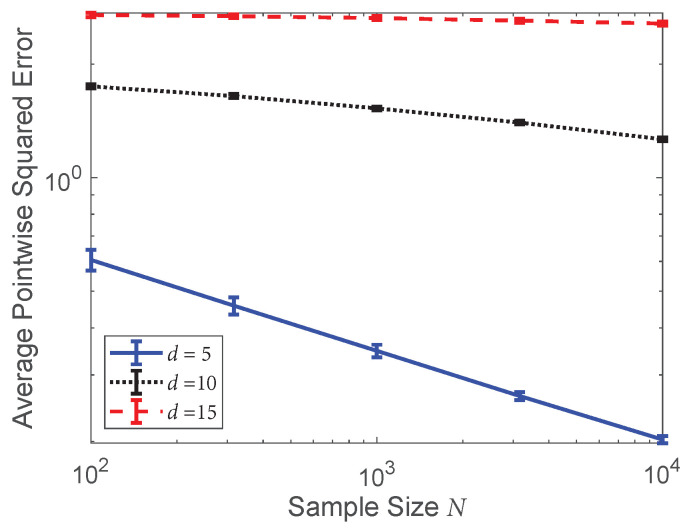
Log–log plot of the average pointwise squared error between the KDE ${\tilde{f}}_{1,h}$ and $f_{1}$ for various dimensions and sample sizes using the same bandwidth and kernel as the standard plug-in estimators in Figure 3. The KDE and the density were compared at 10,000 points sampled from $f_{1}$.

To illustrate the difference between the problems of density estimation and divergence functional estimation, we estimated the average pointwise squared error between the KDE ${\tilde{f}}_{1,h}$ and $f_{1}$ in the previous experiment. We used exactly the same bandwidth and kernel as the standard plug-in estimators in Figure 3 and calculated the pointwise error at 10,000 points sampled from $f_{1}$. The results are shown in Figure 4. From these results, we see that the KDEs performed worse as the dimension of the densities increased. Additionally, we observe by comparing Figure 3 and Figure 4, the average pointwise squared error decreased at a much slower rate as a function of the sample size (*N*) than the MSE of the plug-in divergence estimators, especially for larger dimensions (*d*).

Our experiments indicated that the proposed ensemble estimator is not sensitive to the tuning parameters. See reference [72] for more details.

### 4.3. Central Limit Theorem Validation: KL Divergence

**Figure 5:**
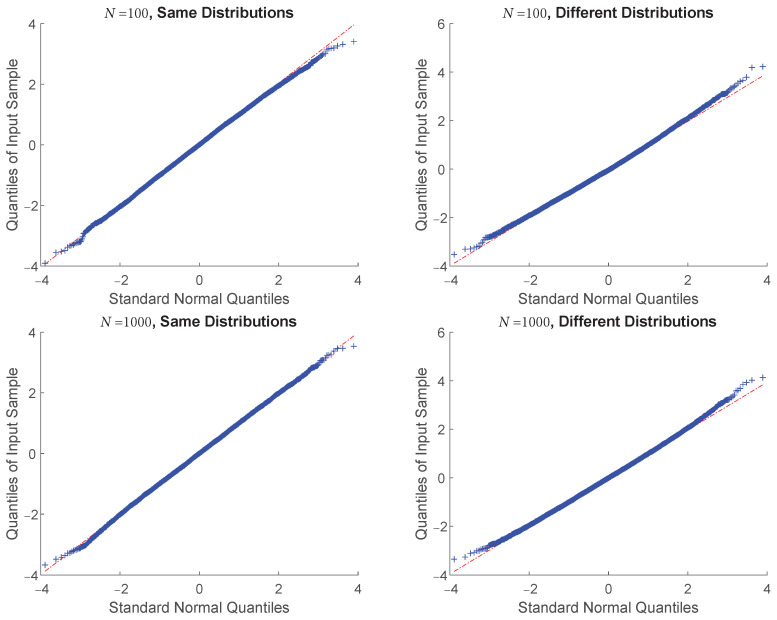
QQ-plots comparing the quantiles of a standard normal random variable and the quantiles of the centered and scaled EnDive estimator applied to the Kullback–Leibler (KL) divergence when the distributions were the same and different. Quantiles were computed from 10,000 trials. These plots correspond to the same experiments as in Table 2 when N = 100 and N = 1000. The correspondence between quantiles is high for all cases.

To verify the central limit theorem of the EnDive estimator, we estimated the KL divergence between two truncated Gaussian densities, again restricted to the unit cube. We conducted two experiments where (1) the densities were different with means of ${\bar{\mu }}_{1}=0.7\times {\bar{1}}_{d}$, ${\bar{\mu }}_{2}=0.3\times {\bar{1}}_{d}$ and covariances of matrices $\sigma_{i}\times I_{d}$, $\sigma_{1}=0.1$, $\sigma_{2}=0.3$; and where (2) the densities were the same with means of $0.3\times {\bar{1}}_{d}$ and covariance matrices of $0.3\times I_{d}$. For both experiments, we chose d=6 and four different sample sizes (*N*). We found that the correspondence between the quantiles of the standard normal distribution and the quantiles of the centered and scaled EnDive estimator was very high under all settings (see Table 2 and Figure 5) which validates Theorem 4.

### 4.4. Bayes Error Rate Estimation on Single-Cell Data

**Figure 6:**
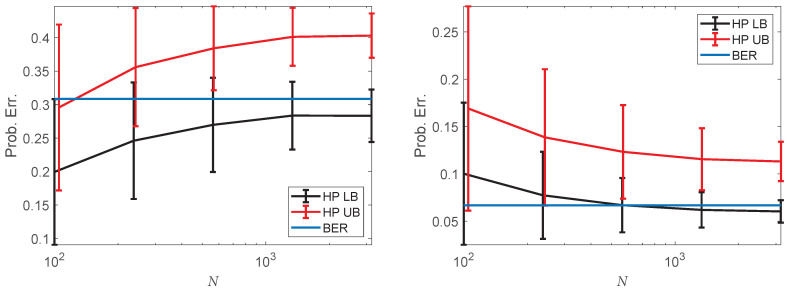
Estimated upper (UB) and lower bounds (LB) on the Bayes error rate (BER) based on estimating the HP divergence between two 10-dimensional Gaussian distributions with identity covariance matrices and distances between means of 1 (**left**) and 3 (**right**), respectively. Estimates were calculated using EnDive, with error bars indicating the standard deviation from 400 trials. The upper bound was closer, on average, to the true BER when *N* was small (≈100–300) and the distance between the means was small. The lower bound was closer, on average, in all other cases.

Using the EnDive estimator, we estimated bounds on the Bayes error rate (BER) of a classification problem involving MARS-seq single-cell RNA-sequencing (scRNA-seq) data measured from developing mouse bone marrow cells enriched for the myeloid and erythroid lineages [73]. However, we first demonstrated the ability of EnDive to estimate the bounds on the BER of a simulated problem. In this simulation, the data were drawn from two classes where each class distribution was a d=10 dimensional Gaussian distribution with different means and the identity covariance matrix. We considered two cases, namely, the distance between the means was 1 or 3. The BER was calculated in both cases. We then estimated upper and lower bounds on the BER by estimating the Henze–Penrose (HP) divergence [4,6]. Figure 6 shows the average estimated upper and lower bounds on the BER with standard error bars for both cases. For all tested sample sizes, the BER was within one standard deviation of the estimated lower bound. The lower bound was also closer, on average, to the BER for most of the tested sample sizes (lower sample sizes with smaller distances between means were the exceptions). Generally, these resuls indicate that the true BER is relatively close to the estimated lower bound, on average.

We then estimated similar bounds on the scRNA-seq classification problem using EnDive. We considered the three most common cell types within the data: erythrocytes (eryth.), monocytes (mono.), and basophils (baso.) (N=1095,559,300, respectively). We estimated the upper and lower bounds on the pairwise BER between these classes using different combinations of genes selected from the Kyoto Encyclopedia of Genes and Genomes (KEGG) pathways associated with the hematopoietic cell lineage [74,75,76]. Each collection of genes contained 11–14 genes. The upper and lower bounds on the BER were estimated using the Henze–Penrose divergence [4,6]. The standard deviations of the bounds for the KEGG-based genes were estimated via 1000 bootstrap iterations. The KEGG-based bounds were compared to BER bounds obtained from 1000 random selections of 12 genes. In all cases, we compared the bounds to the performance of a quadratic discriminant analysis classifier (QDA) with 10-fold cross validation. Note that to correct for undersampling in scRNA-seq data, we first imputed the undersampled data using MAGIC [77].

All results are given in Table 3. From these results, we note that erythrocytes are relatively easy to distinguish from the other two cell types as the BER lower bounds were within nearly two standard deviations of zero when using genes associated with platelet, erythrocyte, and neutrophil development as well as a random selection of 12 genes. This is corroborated by the QDA cross-validated results which were all within two standard deviations of either the upper or lower bound for these gene sets. In contrast, the macrophage-associated genes seem to be less useful for distinguishing erythrocytes than the other gene sets.

We also found that basophils are difficult to distinguish from monocytes using these gene sets. Assuming the relative abundance of each cell type is representative of the population, a trivial upper bound on the BER is 300/(300+559)≈0.35 which is between all of the estimated lower and upper bounds. The QDA results were also relatively high (and may be overfitting the data in some cases based on the estimated BER bounds), suggesting that different genes should be explored for this classification problem.

## 5. Conclusions

We derived the MSE convergence rates for a kernel density plug-in estimator for a large class of divergence functionals. We generalized the theory of optimally weighted ensemble estimation and derived an ensemble divergence estimator EnDive that achieves the parametric rate when the densities are more than *d* times differentiable. The estimator we derived can be applied to general bounded density support sets and can be implemented without knowledge of the support, which is a distinct advantage over other competing estimators. We also derived the asymptotic distribution of the estimator, provided some guidelines for tuning parameter selection, and experimentally validated the theoretical convergence rates for the case of empirical estimation of the Rényi-α divergence integral. We then performed experiments to examine the estimator’s robustness to the choice of tuning parameters, validated the central limit theorem for KL divergence estimation, and estimated bounds on the Bayes error rate for a single cell classification problem.

We note that based on the proof techniques employed in our work, our weighted ensemble estimators are easily extended beyond divergence estimation to more general distributional functionals which may be integral functionals of any number of probability distributions. We also show in Appendix B that EnDive can be easily modified to obtain an estimator that achieves the parametric rate when the densities are more than d/2 times differentiable and the functional *g* has a specific form that includes the Rényi and KL divergences. Future work includes extending this modification to functionals with more general forms. An important divergence of interest in this context is the Henze–Penrose divergence that we used to bound the Bayes error. Further future work will focus on extending this work on divergence estimation to *k*-nn based estimators where knowledge of the support is, again, not required. This will improve the computational burden, as *k*-nn estimators require fewer computations than standard KDEs.

## Figures and Tables

**Table 1:** Negative log–log slope of the EnDive mean squared error (MSE) as a function of the sample size for various dimensions. The slope was calculated beginning at $N_{start}$. The negative slope was closer to 1 with $N_{start}=10^{2.375}$ than for $N_{start}=10^{2}$ indicating that the asymptotic rate had not yet taken effect at $N_{start}=10^{2}$.

Estimator	d=5	d=10	d=15
$N_{start}=10^{2}$	0.85	0.84	0.80
$N_{start}=10^{2.375}$	0.96	0.96	0.95

**Table 2:** Comparison between quantiles of a standard normal random variable and the quantiles of the centered and scaled EnDive estimator applied to the KL divergence when the distributions were the same and different. Quantiles were computed from 10,000 trials. The parameter ρ gives the correlation coefficient between the quantiles, while β is the estimated slope between the quantiles. The correspondence between quantiles was very high for all cases.

N	Same		Different	
	1−ρ	β	1−ρ	β
100	$2.35\times 10^{-4}$	1.014	$9.97\times 10^{-4}$	0.993
500	$9.48\times 10^{-5}$	1.007	$5.06\times 10^{-4}$	0.999
1000	$8.27\times 10^{-5}$	0.996	$4.30\times 10^{-4}$	0.988
5000	$8.59\times 10^{-5}$	0.995	$4.47\times 10^{-4}$	1.005

**Table 3:** Misclassification rate of a quadratic discriminant analysis classifier (QDA) classifier and estimated upper bounds (UB) and lower bounds (LB) of the pairwise BER between mouse bone marrow cell types using the Henze–Penrose divergence applied to different combinations of genes selected from the KEGG pathways associated with the hematopoietic cell lineage. Results are presented as percentages in the form of mean ± standard deviation. Based on these results, erythrocytes are relatively easy to distinguish from the other two cell types using these gene sets.

	Platelets	Erythrocytes	Neutrophils	Macrophages	Random
Eryth. vs. Mono., LB	2.8±1.5	1.2±0.6	0.6±0.6	8.5±1.2	14.4±8.4
Eryth. vs. Mono., UB	5.3±2.9	2.4±1.3	1.2±1.3	15.5±1.9	23.2±12.3
Eryth. vs. Mono., Prob. Error	0.9	0.4	1.3	3.4	7.2±5.4
Eryth. vs. Baso., LB	0.5±0.6	0.05±0.12	0.6±0.5	5.1±0.9	11.9±5.5
Eryth. vs. Baso., UB	1.0±1.1	0.1±0.2	1.1±0.9	9.6±1.6	20.3±8.8
Eryth. vs. Baso., Prob. Error	1.2	0.3	1.9	3.6	6.8±5.0
Baso. vs. Mono., LB	31.1±1.8	27.8±3.1	27.1±2.6	31.6±1.3	32.1±2.6
Baso. vs. Mono., UB	42.8±1.4	39.9±2.8	39.4±2.4	43.2±1.0	43.5±1.2
Baso. vs. Mono., Prob. Error	28.8	30.9	23.9	22.4	29.7±5.7

## Abbreviations

The following abbreviations are used in this manuscript:

KL	Kullback–Leibler
MSE	Mean squared error
SVM	Support vector machine
KDE	kernel density estimator
EnDive	Ensemble Divergence
BER	Bayes error rate
scRNA-seq	Single-cell RNA-sequencing
HP	Henze–Penrose

## Appendix A. Bias Assumptions

Our full assumptions to prove the bias expressions for the estimator ${\tilde{G}}_{h}$ were as follows:

- (A.0): Assume that the kernel *K* is symmetric, is a product kernel, and has bounded support in each dimension.
- (A.1): Assume there exist constants $\epsilon_{0},\epsilon_{\infty }$, such that $0<\epsilon_{0}\leq f_{i}\left(x\right)\leq \epsilon_{\infty }<\infty ,\;\forall x\in S.$
- (A.2): Assume that the densities are $f_{i}\in \Sigma \left(s,K_{H}\right)$ in the interior of S with s≥2.
- (A.3): Assume that *g* has an infinite number of mixed derivatives.
- (A.4): Assume that $\left|\frac{\partial^{k+l}g\left(x,y\right)}{\partial x^{k}\partial y^{l}}\right|$, k,l=0,1,… are strictly upper bounded for $\epsilon_{0}\leq x,y\leq \epsilon_{\infty }$.
- (A.5): Assume the following boundary smoothness condition: Let $p_{x}\left(u\right):R^{d}\to R$ be a polynomial in *u* of order $q\leq r=\left\lfloor s\right\rfloor$ whose coefficients are a function of *x* and are r−q times differentiable. Then, assume that
  $$\int_{x\in S}{\left(\int_{u:K(u)>0,\;x+uh\notin S}K\left(u\right)p_{x}\left(u\right)du\right)}^{t}dx=v_{t}\left(h\right),$$
  where $v_{t}\left(h\right)$ admits the expansion
  $$v_{t}\left(h\right)=\sum_{i=1}^{r-q}e_{i,q,t}h^{i}+o\left(h^{r-q}\right),$$
  for some constants $e_{i,q,t}$.

We focused on finite support kernels for simplicity in the proofs, although it is likely that our results extend to some infinitely supported kernels as well. We assumed relatively strong conditions on the smoothness of *g* in A.3 to enable us to obtain an estimator that achieves good convergence rates without knowledge of the boundary of the support set. While this smoothness condition may seem restrictive, in practice, nearly all divergence and entropy functionals of interest satisfy this condition. Functionals of interest that do not satisfy this assumption (e.g., the total variation distance) typically have at least one point that is not differentiable which violates the assumptions of all competing estimators [54,57,58,59,60,65]. We also note that to obtain simply an upper bound on the bias for the plug-in estimator, much less restrictive assumptions on the functional *g* are sufficient.

Assumption A.5 requires the boundary of the density support set to be smooth with respect to the kernel (K(u)) in the sense that the expectation of the area outside of S with respect to any random variable *u* with smooth distribution is a smooth function of the bandwidth (*h*). Note that we do not require knowledge of the support of the unknown densities to actually implement the estimator (${\tilde{G}}_{h}$). As long as assumptions A.0–A.5 are satisfied, then the bias results we obtain are valid, and therefore, we can obtain the parametric rate with the EnDive estimator. This is in contrast to many other estimators of information theoretic measures such as those presented in references [59,60,65]. In these cases, the boundary of the support set must be known precisely to perform boundary correction to obtain the parametric rate, since the boundary correction is an explicit step in these estimators. In contrast, we do not need to explicitly perform a boundary correction.

It is not necessary for the boundary of S to have smooth contours with no edges or corners as assumption A.5 is satisfied by the following case:

**Theorem** **A1.**
*Assumption A.5 is satisfied when $S={\left[-1,1\right]}^{d}$ and when K is the uniform rectangular kernel; that is, K(x)=1 for all ${x:\;||x||}_{1}\leq 1/2$.*

The proof is given in Appendix D. The methods used to prove this can be easily extended to show that A.5 is satisfied with the uniform rectangular kernel and other similar supports with flat surfaces and corners. Furthermore, we showed in reference [78] that A.5 is satisfied using the uniform spherical kernel with a density support set equal to the unit cube. Note that assumption A.0 is trivially satisfied by the uniform rectangular kernel as well. Again, this is easily extended to more complicated density support sets that have boundaries that contain flat surfaces and corners. Determining other combinations of kernels and density support sets that satisfy A.5 is left for future work.

Densities for which assumptions A.1–A.2 hold include the truncated Gaussian distribution and the beta distribution on the unit cube. Functions for which assumptions A.3–A.4 hold include $g\left(x,y\right)=-\ln\left(\frac{x}{y}\right)$ and $g\left(x,y\right)={\left(\frac{x}{y}\right)}^{\alpha }.$

## Appendix B. Modified EnDive

If the functional *g* has a specific form, we can modify the EnDive estimator to obtain an estimator that achieves the parametric rate when s>d/2. Specifically, we have the following theorem:

**Theorem** **A2.** *Assume that assumptions A.0–A.5* hold. Furthermore, if g(x,y)*has k,l-th order mixed derivatives $\frac{\partial^{k+l}g\left(x,y\right)}{\partial x^{k}\partial y^{l}}$ that depend on x,y only through $x^{\alpha }y^{\beta }$ for some α,β∈R, then for any positive integer λ≥2, the bias of ${\tilde{G}}_{h}$ is*

(A1) $$\begin{array}{ccc} B\left[{\tilde{G}}_{h}\right] & = & \sum_{j=1}^{\left\lfloor s\right\rfloor }c_{10,j}h^{j}+\sum_{q=1}^{\lambda /2}\sum_{j=0}^{\left\lfloor s\right\rfloor }c_{11,q,j}\frac{h^{j}}{{\left(Nh^{d}\right)}^{q}}\\ & & +O\left(h^{s}+\frac{1}{{\left(Nh^{d}\right)}^{\frac{\lambda }{2}}}\right). \end{array}$$

Divergence functionals that satisfy the mixed derivatives condition required for (A1) include the KL divergence and the Rényi-α divergence. Obtaining similar terms for other divergence functionals requires us to separate the dependence on *h* of the derivatives of *g* evaluated at $E_{Z}{\tilde{f}}_{i,h}\left(Z\right)$. This is left for future work. See Appendix E for details.

As compared to (3), there are many more terms in (A1). These terms enable us to modify the EnDive estimator to achieve the parametric MSE convergence rate when s>d/2 for an appropriate choice of bandwidths, whereas the terms in (3) requires s≥d to achieve the same rate. This is accomplished by letting h(l) decrease at a faster rate, as follows.

Let δ>0 and $h\left(l\right)=lN^{\frac{-1}{d+\delta }}$ where l∈L. The bias of each estimator in the resulting ensemble has terms proportional to $l^{j-dq}N^{-\frac{j+q}{d+\delta }}$, where j,q≥0 and j+q>0. Then, the bias of ${\tilde{G}}_{h(l)}$ satisfies condition C.1 if $\phi_{j,q,d}\left(N\right)=N^{-\frac{j+q}{d+\delta }}$, $\psi_{j,q}\left(l\right)=l^{j-dq}$, and

(A2) $$J=\left\{\left\{j,q\right\}:0<j+q<\left(d+1\right)/2,\;q\in \left\{0,1,2,\ldots ,\lambda /2\right\},\;j\in \left\{0,1,2,\ldots ,\left\lfloor s\right\rfloor \right\}\right\},$$

as long as L>|J|=I. The variance also satisfies condition C.2. The optimal weight ($w_{0}$) is found by using (6) to obtain an optimally weighted plug-in divergence functional estimator ${\tilde{G}}_{w_{0}}$ that achieves the parametric convergence rate if λ≥d/δ+1 and if s≥(d+δ)/2. Otherwise, if s<(d+δ)/2, we can only guarantee the MSE rate up to $O\left(\frac{1}{N^{2s/(d+\delta )}}\right)$. We refer to this estimator as the modified EnDive estimator and denote it as ${\tilde{G}}_{\mathrm{Mod}}$. The ensemble estimator ${\tilde{G}}_{\mathrm{Mod}}$ is summarized in Algorithm 1 when δ=1.

**Algorithm A1:** The Modified EnDive Estimator
Input: η, *L* positive real numbers L, samples $\left\{Y_{1},\ldots ,Y_{N}\right\}$ from $f_{1}$, samples $\left\{X_{1},\ldots ,X_{N}\right\}$ from $f_{2}$, dimension *d*, function *g*, kernel *K* Output: The modified EnDive estimator ${\tilde{G}}_{\mathrm{Mod}}$ 1: Solve for $w_{0}$ using (6) with $\phi_{j,q,d}\left(N\right)=N^{-\frac{j+q}{d+1}}$ and basis functions $\psi_{j,q}\left(l\right)=l^{j-dq}$, $l\in \bar{l}$, and {i,j}∈J defined in (A2) 2: **for all** $l\in \bar{l}$ **do** 3: $h\left(l\right)\leftarrow lN^{\frac{-1}{d+1}}$ 4: **for** i=1 to *N* **do** 5: ${\tilde{f}}_{1,h(l)}\left(X_{i}\right)\leftarrow \frac{1}{Nh{\left(l\right)}^{d}}\sum_{j=1}^{N}K\left(\frac{X_{i}-Y_{j}}{h(l)}\right)$, ${\tilde{f}}_{2,h(l)}\left(X_{i}\right)\leftarrow \frac{1}{\left(N-1\right)h{\left(l\right)}^{d}}\sum_{\begin{array}{c} j=1j\neq i \end{array}}^{N}K\left(\frac{X_{i}-X_{j}}{h(l)}\right)$ 6: **end for** 7: ${\tilde{G}}_{h(l)}\leftarrow \frac{1}{N}\sum_{i=1}^{N}g\left({\tilde{f}}_{1,h(l)}\left(X_{i}\right),{\tilde{f}}_{2,h(l)}\left(X_{i}\right)\right)$ 8: **end for** 9: ${\tilde{G}}_{\mathrm{Mod}}\leftarrow \sum_{l\in L}w_{0}\left(l\right){\tilde{G}}_{h(l)}$

The parametric rate can be achieved with ${\tilde{G}}_{\mathrm{Mod}}$ under less strict assumptions on the smoothness of the densities than those required for ${\tilde{G}}_{\mathrm{EnDive}}$. Since δ>0 can be arbitrary, it is theoretically possible to achieve the parametric rate with the modified estimator as long as s>d/2. This is consistent with the rate achieved by the more complex estimators proposed in reference [57]. We also note that the central limit theorem applies and that the convergence is uniform as Theorem 5 applies for $s>\left\lfloor (d+\delta )/2\right\rfloor$ and s≥(d+δ)/2.

These rate improvements come at a cost for the number of parameters (*L*) required to implement the weighted ensemble estimator. If $s\geq \frac{d+\delta }{2}$, then the size of *J* for ${\tilde{G}}_{\mathrm{Mod}}$ is in the order of $\frac{d^{2}}{8\delta }$. This may lead to increased variance in the ensemble estimator as indicated by (5).

So far, ${\tilde{G}}_{\mathrm{Mod}}$ can only be applied to functionals (g(x,y)) with mixed derivatives of the form of $x^{\alpha }y^{\beta }$. Future work is required to extend this estimator to other functionals of interest.

## Appendix C. General Results

Here we present the generalized forms of Theorems 1 and 2 where the sample sizes and bandwidths of the two datasets are allowed to differ. In this case, the KDEs are

$$\begin{array}{ccc} {\tilde{f}}_{1,h_{1}}\left(X_{j}\right) & = & \frac{1}{N_{1}h_{1}^{d}}\sum_{i=1}^{N_{1}}K\left(\frac{X_{j}-Y_{i}}{h_{1}}\right),\\ {\tilde{f}}_{2,h_{2}}\left(X_{j}\right) & = & \frac{1}{M_{2}h_{2}^{d}}\sum_{\begin{array}{c} i=1\\ i\neq j \end{array}}^{N_{2}}K\left(\frac{X_{j}-X_{i}}{h_{2}}\right), \end{array}$$

where $M_{2}=N_{2}-1$. $G\left(f_{1},f_{2}\right)$ is then approximated as

(A3) $${\tilde{G}}_{h_{1},h_{2}}=\frac{1}{N_{2}}\sum_{i=1}^{N_{2}}g\left({\tilde{f}}_{1,h_{1}}\left(X_{i}\right),{\tilde{f}}_{2,h_{2}}\left(X_{i}\right)\right).$$

We also generalize the bias result to the case where the kernel (*K*) has the order ν which means that the *j*-th moment of the kernel $K_{i}$ defined as $\int t^{j}K_{i}\left(t\right)dt$ is zero for all j=1,…,ν−1 and i=1,…,d where $K_{i}$ is the kernel in the *i*-th coordinate. Note that symmetric product kernels have the order ν≥2. The following theorem on the bias follows under assumptions A.0–A.5:

**Theorem** **A3.**
*For general g, the bias of the plug-in estimator (${\tilde{G}}_{h_{1},h_{2}}$) is of the form*

(A4) $$\begin{array}{ccc} B\left[{\tilde{G}}_{h_{1},h_{2}}\right] & = & \sum_{j=1}^{r}\left(c_{4,1,j}h_{1}^{j}+c_{4,2,j}h_{2}^{j}\right)+\sum_{j=1}^{r}\sum_{i=1}^{r}c_{5,i,j}h_{1}^{j}h_{2}^{i}+O\left(h_{1}^{s}+h_{2}^{s}\right)\\ & & +c_{9,1}\frac{1}{N_{1}h_{1}^{d}}+c_{9,2}\frac{1}{N_{2}h_{2}^{d}}+o\left(\frac{1}{N_{1}h_{1}^{d}}+\frac{1}{N_{2}h_{2}^{d}}\right). \end{array}$$

*Furthermore, if g(x,y) has k,l-th order mixed derivatives $\frac{\partial^{k+l}g\left(x,y\right)}{\partial x^{k}\partial y^{l}}$ that depend on x,y only through $x^{\alpha }y^{\beta }$ for some α,β∈R, then for any positive integer λ≥2, the bias is of the form*

(A5) $$\begin{array}{ccc} B\left[{\tilde{G}}_{h_{1},h_{2}}\right] & = & \sum_{j=1}^{r}\left(c_{4,1,j}h_{1}^{j}+c_{4,2,j}h_{2}^{j}\right)+\sum_{j=1}^{r}\sum_{i=1}^{r}c_{5,i,j}h_{1}^{j}h_{2}^{i}+O\left(h_{1}^{s}+h_{2}^{s}\right)\\ & & \sum_{j=1}^{\lambda /2}\sum_{m=0}^{r}\left(c_{9,1,j,m}\frac{h_{1}^{m}}{{\left(N_{1}h_{1}^{d}\right)}^{j}}+c_{9,2,j,m}\frac{h_{2}^{m}}{{\left(N_{2}h_{2}^{d}\right)}^{j}}\right)\\ & & +\sum_{j=1}^{\lambda /2}\sum_{m=0}^{r}\sum_{i=1}^{\lambda /2}\sum_{n=0}^{r}c_{9,j,i,m,n}\frac{h_{1}^{m}h_{2}^{n}}{{\left(N_{1}h_{1}^{d}\right)}^{j}{\left(N_{2}h_{2}^{d}\right)}^{i}}\\ & & +O\left(\frac{1}{{\left(N_{1}h_{1}^{d}\right)}^{\frac{\lambda }{2}}}+\frac{1}{{\left(N_{2}h_{2}^{d}\right)}^{\frac{\lambda }{2}}}\right). \end{array}$$

Note that the bandwidth and sample size terms do not depend on the order of the kernel (ν). Thus, using a higher-order kernel does not provide any benefit to the convergence rates. This lack of improvement is due to the bias of the density estimators at the boundary of the density support sets. To obtain better convergence rates using higher-order kernels, boundary correction would be necessary [57,60]. In contrast, we improve the convergence rates by using a weighted ensemble that does not require boundary correction.

The variance result requires much less strict assumptions than the bias results:

**Theorem** **A4.** *Assume that the functional**g* in *(1) is Lipschitz continuous in both of its arguments with the Lipschitz constant $C_{g}$. Then, the variance of the plug-in estimator (${\tilde{G}}_{h_{1},h_{2}}$) is bounded by*

$$V\left[{\tilde{G}}_{h_{1},h_{2}}\right]\leq C_{g}^{2}{\left||K|\right|}_{\infty }^{2}\left(\frac{10}{N_{2}}+\frac{N_{1}}{N_{2}^{2}}\right).$$

The proofs of these theorems are in Appendix E and Appendix F. Theorems 1 and 2 then follow.

## Appendix D. Proof of Theorem A1 (Boundary Conditions)

Consider a uniform rectangular kernel K(x) that satisfies K(x)=1 for all *x*, such that ${\left||x|\right|}_{1}\leq 1/2$. Also, consider the family of probability densities (*f*) with rectangular support $S={\left[-1,1\right]}^{d}$. We prove Theorem A1 which is that that S satisfies the following smoothness condition (A.5): for any polynomial $p_{x}\left(u\right):R^{d}\to R$ of order $q\leq r=\left\lfloor s\right\rfloor$ with coefficients that are r−q times differentiable wrt *x*,

(A6) $$\int_{x\in S}{\left(\int_{{u:||u||}_{1}\leq \frac{1}{2},\;x+uh\notin S}p_{x}\left(u\right)du\right)}^{t}dx=v_{t}\left(h\right),$$

where $v_{t}\left(h\right)$ has the expansion

$$v_{t}\left(h\right)=\sum_{i=1}^{r-q}e_{i,q,t}h^{i}+o\left(h^{r-q}\right).$$

Note that the inner integral forces the *x*s under consideration to be boundary points via the constraint x+uh∉S.

### Appendix D.1. Single Coordinate Boundary Point

We begin by focusing on points *x* that are boundary points by virtue of a single coordinate $x_{i}$, such that $x_{i}+u_{i}h\notin S$. Without loss of generality, assume that $x_{i}+u_{i}h>1$. The inner integral in (A6) can then be evaluated first with respect to (wrt) all coordinates other than *i*. Since all of these coordinates lie within the support, the inner integral over these coordinates will amount to integration of the polynomial $p_{x}\left(u\right)$ over a symmetric d−1 dimensional rectangular region $\left|u_{j}\right|\leq \frac{1}{2}$ for all j≠i. This yields a function $\sum_{m=1}^{q}{\tilde{p}}_{m}\left(x\right)u_{i}^{m}$ where the coefficients ${\tilde{p}}_{m}\left(x\right)$ are each r−q times differentiable wrt *x*.

With respect to the $u_{i}$ coordinate, the inner integral will have limits from $\frac{1-x_{i}}{h}$ to $\frac{1}{2}$ for some $1>x_{i}>1-\frac{h}{2}$. Consider the ${\tilde{p}}_{q}\left(x\right)u_{i}^{q}$ monomial term. The inner integral wrt this term yields

(A7) $$\sum_{m=1}^{q}{\tilde{p}}_{m}\left(x\right)\int_{\frac{1-x_{i}}{h}}^{\frac{1}{2}}u_{i}^{m}du_{i}=\sum_{m=1}^{q}{\tilde{p}}_{m}\left(x\right)\frac{1}{m+1}\left(\frac{1}{2^{m+1}}-{\left(\frac{1-x_{i}}{h}\right)}^{m+1}\right).$$

Raising the right-hand-side of (A7) to the power of *t* results in an expression of the form

(A8) $$\sum_{j=0}^{qt}{\overset{ˇ}{p}}_{j}\left(x\right){\left(\frac{1-x_{i}}{h}\right)}^{j},$$

where the coefficients ${\overset{ˇ}{p}}_{j}\left(x\right)$ are r−q times differentiable wrt *x*. Integrating (A8) over all the coordinates in *x* other than $x_{i}$ results in an expression of the form

(A9) $$\sum_{j=0}^{qt}{\bar{p}}_{j}\left(x_{i}\right){\left(\frac{1-x_{i}}{h}\right)}^{j},$$

where, again, the coefficients ${\bar{p}}_{j}\left(x_{i}\right)$ are r−q times differentiable wrt $x_{i}$. Note that since the other coordinates of *x* other than $x_{i}$ are far away from the boundary, the coefficients ${\bar{p}}_{j}\left(x_{i}\right)$ are independent of *h*. To evaluate the integral of (A9), consider the r−q term Taylor series expansion of ${\bar{p}}_{j}\left(x_{i}\right)$ around $x_{i}=1$. This will yield terms of the form

$$\begin{array}{ccc} \int_{1-h/2}^{1}\frac{{\left(1-x_{i}\right)}^{j+k}}{h^{k}}dx_{i} & = & {\left.-\frac{{\left(1-x_{i}\right)}^{j+k+1}}{h^{k}\left(j+k+1\right)}\right|}_{x_{i}=1-h/2}^{x_{i}=1}\\ & = & \frac{h^{j+1}}{\left(j+k+1\right)2^{j+k+1}}, \end{array}$$

for 0≤j≤r−q, and 0≤k≤qt. Combining terms results in the expansion $v_{t}\left(h\right)=\sum_{i=1}^{r-q}e_{i,q,t}h^{i}+o\left(h^{r-q}\right)$.

### Appendix D.2. Multiple Coordinate Boundary Point

The case where multiple coordinates of point *x* are near the boundary is a straightforward extension of the single boundary point case, so we only sketch the main ideas here. As an example, consider the case where two of the coordinates are near the boundary. Assume for notational ease that they are $x_{1}$ and $x_{2}$ and that $x_{1}+u_{1}h>1$ and $x_{2}+u_{2}h>1$. The inner integral in (A6) can again be evaluated first wrt all coordinates other than 1 and 2. This yields a function $\sum_{m,j=1}^{q}{\tilde{p}}_{m,j}\left(x\right)u_{1}^{m}u_{2}^{j}$ where the coefficients ${\tilde{p}}_{m,j}\left(x\right)$ are each r−q times differentiable wrt *x*. Integrating this wrt $x_{1}$ and $x_{2}$ and then raising the result to the power of *t* yields a double sum similar to (A8). Integrating this over all the coordinates in *x* other than $x_{1}$ and $x_{2}$ gives a double sum similar to (A9). Then, a Taylor series expansion of the coefficients and integration over $x_{1}$ and $x_{2}$ yields the result.

## Appendix E. Proof of Theorem A3 (Bias)

In this appendix, we prove the bias results in Theorem A3. The bias of the base kernel density plug-in estimator ${\tilde{G}}_{h_{1},h_{2}}$ can be expressed as

(A10) $$\begin{array}{ccc} B\left[{\tilde{G}}_{h_{1},h_{2}}\right] & = & E\left[g\left({\tilde{f}}_{1,h_{1}}\left(Z\right),{\tilde{f}}_{2,h_{2}}\left(Z\right)\right)-g\left(f_{1}\left(Z\right),f_{2}\left(Z\right)\right)\right]\\ & = & E\left[g\left({\tilde{f}}_{1,h_{1}}\left(Z\right),{\tilde{f}}_{2,h_{2}}\left(Z\right)\right)-g\left(E_{Z}{\tilde{f}}_{1,h_{1}}\left(Z\right),E_{Z}{\tilde{f}}_{2,h_{2}}\left(Z\right)\right)\right]\\ & & +E\left[g\left(E_{Z}{\tilde{f}}_{1,h_{1}}\left(Z\right),E_{Z}{\tilde{f}}_{2,h_{2}}\left(Z\right)\right)-g\left(f_{1}\left(Z\right),f_{2}\left(Z\right)\right)\right], \end{array}$$

where Z is drawn from $f_{2}$. The first term is the “variance” term, while the second is the “bias” term. We bound these terms using Taylor series expansions under the assumption that *g* is infinitely differentiable. The Taylor series expansion of the variance term in (A11) will depend on variance-like terms of the KDEs, while the Taylor series expansion of the bias term in (A11) will depend on the bias of the KDEs.

The Taylor series expansion of $g\left(E_{Z}{\tilde{f}}_{1,h_{1}}\left(Z\right),E_{Z}{\tilde{f}}_{2,h_{2}}\left(Z\right)\right)$ around $f_{1}\left(Z\right)$ and $f_{2}\left(Z\right)$ is

(A11) $$\begin{array}{c} g\left(E_{Z}{\tilde{f}}_{1,h_{1}}\left(Z\right),E_{Z}{\tilde{f}}_{2,h_{2}}\left(Z\right)\right)=\sum_{i=0}^{\infty }\sum_{j=0}^{\infty }\left({\left.\frac{\partial^{i+j}g\left(x,y\right)}{\partial x^{i}\partial y^{j}}\right|}_{\begin{array}{c} x=f_{1}\left(Z\right)\\ y=f_{2}\left(Z\right) \end{array}}\right)\frac{B_{Z}^{i}\left[{\tilde{f}}_{1,h_{1}}\left(Z\right)\right]B_{Z}^{j}\left[{\tilde{f}}_{2,h_{2}}\left(Z\right)\right]}{i!j!}, \end{array}$$

where $B_{Z}^{j}\left[{\tilde{f}}_{i,h_{i}}\left(Z\right)\right]={\left(E_{Z}{\tilde{f}}_{i,h_{i}}\left(Z\right)-f_{i}\left(Z\right)\right)}^{j}$ is the bias of ${\tilde{f}}_{i,h_{i}}$ at the point Z raised to the power of *j*. This expansion can be used to control the second term (the bias term) in (A11). To accomplish this, we require an expression for $E_{Z}{\tilde{f}}_{i,h_{i}}\left(Z\right)-f_{i}\left(Z\right)=B_{Z}\left[{\tilde{f}}_{i,h_{i}}\left(Z\right)\right]$.

To obtain an expression for $B_{Z}\left[{\tilde{f}}_{i,h_{i}}\left(Z\right)\right]$, we separately consider the cases when Z is in the interior of the support S or when Z is near the boundary of the support. A point X∈S is defined to be in the interior of S if for all Y∉S, $K\left(\frac{X-Y}{h_{i}}\right)=0$. A point X∈S is near the boundary of the support if it is not in the interior. Denote the region in the interior and near the boundary wrt $h_{i}$ as $S_{I_{i}}$ and $S_{B_{i}}$, respectively. We will need the following:

**Lemma** **A1.**
*Let Z be a realization of the density $f_{2}$ independent of ${\tilde{f}}_{i,h_{i}}$ for i=1,2. Assume that the densities $f_{1}$ and $f_{2}$ belong to Σ(s,L). Then, for $Z\in S_{I_{i}}$,*

(A12) $$E_{Z}\left[{\tilde{f}}_{i,h_{i}}\left(Z\right)\right]=f_{i}\left(Z\right)+\sum_{j=\nu /2}^{\left\lfloor s/2\right\rfloor }c_{i,j}\left(Z\right)h_{i}^{2j}+O\left(h_{i}^{s}\right).$$

**Proof.** Obtaining the lower order terms in (A12) is a common result in kernel density estimation. However, since we also require the higher order terms, we present the proof here. Additionally, some of the results in this proof will be useful later. From the linearity of the KDE, we have that if X is drawn from $f_{i}$ and is independent of Z, then

(A13) $$\begin{array}{ccc} E_{Z}{\tilde{f}}_{i,h_{i}}\left(Z\right) & = & E_{Z}\left[\frac{1}{h_{i}^{d}}K\left(\frac{X-Z}{h_{i}}\right)\right]\\ & = & \int \frac{1}{h_{i}^{d}}K\left(\frac{x-Z}{h_{i}}\right)f_{i}\left(x\right)dx\\ & = & \int K\left(t\right)f_{i}\left(th_{i}+Z\right)dt, \end{array}$$

where the last step follows on from the substitution $t=\frac{x-Z}{h_{i}}$. Since the density ($f_{i}$) belongs to Σ(s,K), by using multi-index notation we can expand it to

(A14) $$f_{i}\left(th_{i}+Z\right)=f_{i}\left(Z\right)+\sum_{0<|\alpha |\leq \left\lfloor s\right\rfloor }\frac{D^{\alpha }f_{i}\left(Z\right)}{\alpha !}{\left(th_{i}\right)}^{\alpha }+O\left({∥th_{i}∥}^{s}\right),$$

where $\alpha !=\alpha_{1}!\alpha_{2}!\ldots \alpha_{d}!$ and $Y^{\alpha }=Y_{1}^{\alpha_{1}}Y_{2}^{\alpha_{2}}\ldots Y_{d}^{\alpha_{d}}$. Combining (A13) and (A14) gives

$$\begin{array}{ccc} E_{Z}{\tilde{f}}_{i,h_{i}}\left(Z\right) & = & f_{i}\left(Z\right)+\sum_{0<|\alpha |\leq \left\lfloor s\right\rfloor }\frac{D^{\alpha }f_{i}\left(Z\right)}{\alpha !}h_{i}^{\left|\alpha \right|}\int t^{\alpha }K\left(t\right)dt+O\left(h_{i}^{s}\right)\\ & = & f_{i}\left(Z\right)+\sum_{j=\nu /2}^{\left\lfloor s/2\right\rfloor }c_{i,j}\left(Z\right)h_{i}^{2j}+O\left(h_{i}^{s}\right), \end{array}$$

where the last step follows from the fact that *K* is symmetric and of order ν. ☐

To obtain a similar result for the case when Z is near the boundary of S, we use the assumption A.5.

**Lemma** **A2.**
*Let γ(x,y) be an arbitrary function satisfying $\sup_{x,y}\left|\gamma \left(x,y\right)\right|<\infty$. Let S satisfy the boundary smoothness conditions of Assumption A.5. Assume that the densities $f_{1}$ and $f_{2}$ belong to Σ(s,L), and let Z be a realization of the $f_{2}$ density independently of ${\tilde{f}}_{i,h_{i}}$ for i=1,2. Let $h^{{}^{\prime }}=\min\left(h_{1},h_{2}\right)$. Then,*

(A15) $$\begin{array}{ccc} E\left[1_{\left\{Z\in S_{B_{i}}\right\}}\gamma \left(f_{1}\left(Z\right),f_{2}\left(Z\right)\right)B_{Z}^{t}\left[{\tilde{f}}_{i,h_{i}}\left(Z\right)\right]\right] & = & \sum_{j=1}^{r}c_{4,i,j,t}h_{i}^{j}+o\left(h_{i}^{r}\right) \end{array}$$

(A16) $$\begin{array}{ccc} E\left[1_{\left\{Z\in S_{B_{1}}\cap S_{B_{2}}\right\}}\gamma \left(f_{1}\left(Z\right),f_{2}\left(Z\right)\right)B_{Z}^{t}\left[{\tilde{f}}_{1,h_{1}}\left(Z\right)\right]B_{Z}^{q}\left[{\tilde{f}}_{2,h_{2}}\left(Z\right)\right]\right] & = & \sum_{j=0}^{r-1}\sum_{i=0}^{r-1}c_{4,j,i,q,t}h_{1}^{j}h_{2}^{i}h^{{}^{\prime }}+o\left({\left(h^{{}^{\prime }}\right)}^{r}\right) \end{array}$$

**Proof.** For a fixed *X* near the boundary of S, we have

$$\begin{array}{ccc} E\left[{\tilde{f}}_{i,h_{i}}\left(X\right)\right]-f_{i}\left(X\right) & = & \frac{1}{h_{i}^{d}}\int_{Y:Y\in S}K\left(\frac{X-Y}{h_{i}}\right)f_{i}\left(Y\right)dY-f_{i}\left(X\right)\\ & = & \left[\frac{1}{h_{i}^{d}}\int_{Y:K\left(\frac{X-Y}{h_{i}}\right)>0}K\left(\frac{X-Y}{h_{i}}\right)f_{i}\left(Y\right)dY-f_{i}\left(X\right)\right]\\ & & -\left[\frac{1}{h_{i}^{d}}\int_{Y:Y\notin S}K\left(\frac{X-Y}{h_{i}}\right)f_{i}\left(Y\right)dY\right]\\ & = & T_{1,i}\left(X\right)-T_{2,i}\left(X\right). \end{array}$$

Note that, in $T_{1,i}\left(X\right)$, we are extending the integral beyond the support of the $f_{i}$ density. However, by using the same Taylor series expansion method as in the proof of Lemma A1, we always evaluate $f_{i}$ and its derivatives at point *X* which is within the support of $f_{i}$. Thus, it does not matter how we define an extension of $f_{i}$ since the Taylor series will remain the same. Thus, $T_{1,i}\left(X\right)$ results in an identical expression to that obtained from (A12). For the $T_{2,i}\left(X\right)$ term, we expand it using multi-index notation as

$$\begin{array}{ccc} T_{2,i}\left(X\right) & = & \frac{1}{h_{i}^{d}}\int_{Y:Y\notin S}K\left(\frac{X-Y}{h_{i}}\right)f_{i}\left(Y\right)dY\\ & = & \int_{u:h_{i}u+X\notin S,K\left(u\right)>0}K\left(u\right)f_{i}\left(X+h_{i}u\right)du\\ & = & \sum_{|\alpha |\leq r}\frac{h_{i}^{\left|\alpha \right|}}{\alpha !}\int_{u:h_{i}u+X\notin S,K\left(u\right)>0}K\left(u\right)D^{\alpha }f_{i}\left(X\right)u^{\alpha }du+o\left(h_{i}^{r}\right). \end{array}$$

Recognizing that the |α|th derivative of $f_{i}$ is r−|α| times differentiable, we can apply assumption A.5 to obtain the expectation of $T_{2,i}\left(X\right)$ wrt *X*:

$$\begin{array}{ccc} E\left[T_{2,i}\left(X\right)\right] & = & \frac{1}{h_{i}^{d}}\int_{X}\int_{Y:Y\notin S}K\left(\frac{X-Y}{h_{i}}\right)f_{i}\left(Y\right)dYf_{2}\left(X\right)dx\\ & = & \sum_{|\alpha |\leq r}\frac{h_{i}^{\left|\alpha \right|}}{\alpha !}\int_{X}\int_{u:h_{i}u+X\notin S,K\left(u\right)>0}K\left(u\right)D^{\alpha }f_{i}\left(X\right)u^{\alpha }duf_{2}\left(X\right)dX+o\left(h_{i}^{r}\right)\\ & = & \sum_{|\alpha |\leq r}\frac{h_{i}^{\left|\alpha \right|}}{\alpha !}\left[\sum_{1\leq |\beta |\leq r-|\alpha |}e_{\beta ,r-|\alpha |}h_{i}^{\left|\beta \right|}+o\left(h_{i}^{r-|\alpha |}\right)\right]+o\left(h_{i}^{r}\right)\\ & = & \sum_{j=1}^{r}e_{j}h_{i}^{j}+o\left(h_{i}^{r}\right). \end{array}$$

Similarly, we find that

$$\begin{array}{ccc} E\left[{\left(T_{2,i}\left(X\right)\right)}^{t}\right] & = & \frac{1}{h_{i}^{dt}}\int_{X}{\left(\int_{Y:Y\notin S}K\left(\frac{X-Y}{h_{i}}\right)f_{i}\left(Y\right)dY\right)}^{t}f_{2}\left(X\right)dx\\ & = & \int_{X}{\left(\sum_{|\alpha |\leq r}\frac{h_{i}^{\left|\alpha \right|}}{\alpha !}\int_{u:h_{i}u+X\notin S,K\left(u\right)>0}K\left(u\right)D^{\alpha }f_{i}\left(X\right)u^{\alpha }du\right)}^{t}f_{2}\left(X\right)dX\\ & = & \sum_{j=1}^{r}e_{j,t}h_{i}^{j}+o\left(h_{i}^{r}\right). \end{array}$$

Combining these results gives

E1Z∈SBγf1(Z),f2(Z)EZf˜i,hi(Z)−fi(Z)t=Eγf1(Z),f2(Z)T1,i(Z)−T2,i(Z)t=Eγf1(Z),f2(Z)∑j=0ttjT1,i(Z)j−T2,i(Z)t−j=∑j=1rc4,i,j,thij+ohir,

where the constants are functionals of the kernel γ and the densities. The expression in (A16) can be proved in a similar manner. ☐

Applying Lemmas A1 and A2 to (A11) gives

(A17) $$E\left[g\left(E_{Z}{\tilde{f}}_{1,h_{1}}\left(Z\right),E_{Z}{\tilde{f}}_{2,h_{2}}\left(Z\right)\right)-g\left(f_{1}\left(Z\right),f_{2}\left(Z\right)\right)\right]=\sum_{j=1}^{r}\left(c_{4,1,j}h_{1}^{j}+c_{4,2,j}h_{2}^{j}\right)+\sum_{j=0}^{r-1}\sum_{i=0}^{r-1}c_{5,i,j}h_{1}^{j}h_{2}^{i}h^{{}^{\prime }}+o\left(h_{1}^{r}+h_{2}^{r}\right).$$

For the variance term (the first term) in (A10), the truncated Taylor series expansion of $g\left({\tilde{f}}_{1,h_{1}}\left(Z\right),{\tilde{f}}_{2,h_{2}}\left(Z\right)\right)$ around $E_{Z}{\tilde{f}}_{1,h_{1}}\left(Z\right)$ and $E_{Z}{\tilde{f}}_{2,h_{2}}\left(Z\right)$ gives

(A18) $$\begin{array}{ccc} g\left({\tilde{f}}_{1,h_{1}}\left(Z\right),{\tilde{f}}_{2,h_{2}}\left(Z\right)\right) & = & \sum_{i=0}^{\lambda }\sum_{j=0}^{\lambda }\left({\left.\frac{\partial^{i+j}g\left(x,y\right)}{\partial x^{i}\partial y^{j}}\right|}_{\begin{array}{c} x=E_{Z}{\tilde{f}}_{1,h_{1}}\left(Z\right)\\ y=E_{Z}{\tilde{f}}_{2,h_{2}}\left(Z\right) \end{array}}\right)\frac{{\tilde{e}}_{1,h_{1}}^{i}\left(Z\right){\tilde{e}}_{2,h_{2}}^{j}\left(Z\right)}{i!j!}+o\left({\tilde{e}}_{1,h_{1}}^{\lambda }\left(Z\right)+{\tilde{e}}_{2,h_{2}}^{\lambda }\left(Z\right)\right) \end{array}$$

where ${\tilde{e}}_{i,h_{i}}\left(Z\right):={\tilde{f}}_{i,h_{i}}\left(Z\right)-E_{Z}{\tilde{f}}_{i,h_{i}}\left(Z\right)$. To control the variance term in (A11), we thus require expressions for $E_{Z}\left[{\tilde{e}}_{i,h_{i}}^{j}\left(Z\right)\right]$.

**Lemma** **A3.**
*Let Z be a realization of the $f_{2}$ density that is in the interior of the support and is independent of ${\tilde{f}}_{i,h_{i}}$ for i=1,2. Let n(q) be the set of integer divisors of q including 1 but excluding q. Then,*

(A19) $$\begin{array}{ccc} E_{Z}\left[{\tilde{e}}_{i,h_{i}}^{q}\left(Z\right)\right] & = & \left\{\begin{array}{cc} \sum_{j\in n(q)}\frac{1}{{\left(N_{2}h_{2}^{d}\right)}^{q-j}}\sum_{m=0}^{\left\lfloor s/2\right\rfloor }c_{6,i,q,j,m}\left(Z\right)h_{i}^{2m}+O\left(\frac{1}{N_{i}}\right), & q\geq 2\\ 0, & q=1, \end{array}\right. \end{array}$$

(A20) $$\begin{array}{ccc} E_{Z}\left[{\tilde{e}}_{1,h_{1}}^{q}\left(Z\right){\tilde{e}}_{2,h_{2}}^{l}\left(Z\right)\right] & = & \left\{\begin{array}{cc} \left(\sum_{i\in n(q)}\frac{1}{{\left(N_{1}h_{1}^{d}\right)}^{q-i}}\sum_{m=0}^{\left\lfloor s/2\right\rfloor }c_{6,1,q,i,m}\left(Z\right)h_{1}^{2m}\right)\times q, & \;l\geq 2\\ \left(\sum_{j\in n(l)}\frac{1}{{\left(N_{2}h_{2}^{d}\right)}^{l-j}}\sum_{t=0}^{\left\lfloor s/2\right\rfloor }c_{6,2,l,j,t}\left(Z\right)h_{2}^{2t}\right)+O\left(\frac{1}{N_{1}}+\frac{1}{N_{2}}\right),\\ 0, & q=1\;\mathrm{or}\;l=1 \end{array}\right. \end{array}$$

*where $c_{6,i,q,j,m}$ is a functional of $f_{1}$ and $f_{2}.$*

**Proof.** Define the random variable $V_{i}\left(Z\right)=K\left(\frac{X_{i}-Z}{h_{2}}\right)-E_{Z}K\left(\frac{X_{i}-Z}{h_{2}}\right)$. This gives

$$\begin{array}{ccc} {\tilde{e}}_{2,h_{2}}\left(Z\right) & = & {\tilde{f}}_{2,h_{2}}\left(Z\right)-E_{Z}{\tilde{f}}_{2,h_{2}}\left(Z\right)\\ & = & \frac{1}{N_{2}h_{2}^{d}}\sum_{i=1}^{N_{2}}V_{i}\left(Z\right). \end{array}$$

Clearly, $E_{Z}V_{i}\left(Z\right)=0$. From (A13), we have for integer j≥1

$$\begin{array}{ccc} E_{Z}\left[K^{j}\left(\frac{X_{i}-Z}{h_{2}}\right)\right] & = & \int K^{j}\left(t\right)f_{2}\left(th_{2}+Z\right)dt\\ & = & h_{2}^{d}\sum_{m=0}^{\left\lfloor s/2\right\rfloor }c_{3,2,j,m}\left(Z\right)h_{2}^{2m}, \end{array}$$

where the constants $c_{3,2,j,m}$ depend on density $f_{2}$, its derivatives, and the moments of kernel $K^{j}$. Note that since *K* is symmetric, the odd moments of $K^{j}$ are zero for Z in the interior of the support. However, all even moments may now be non-zero since $K^{j}$ may now be non-negative. In accordance with the binomial theorem,

EZVij(Z)=∑k=0jjkEZKkXi−Zh2EZKXi−Zh2j−k=∑k=0jjkh2dOh2d(j−k)∑m=0s/2c3,2,k,m(Z)h22m=h2d∑m=0s/2c3,2,j,m(Z)h22m+Oh2d.

We can use these expressions to simplify $E_{Z}\left[{\tilde{e}}_{2,h_{2}}^{q}\left(Z\right)\right]$. As an example, let q=2. Then, since the $X_{i}s$ are independent,

$$\begin{array}{ccc} E_{Z}\left[{\tilde{e}}_{2,h_{2}}^{2}\left(Z\right)\right] & = & \frac{1}{N_{2}h_{2}^{2d}}E_{Z}V_{i}^{2}\left(Z\right)\\ & = & \frac{1}{N_{2}h_{2}^{d}}\sum_{m=0}^{\left\lfloor s/2\right\rfloor }c_{3,2,2,m}\left(Z\right)h_{2}^{2m}+O\left(\frac{1}{N_{2}}\right). \end{array}$$

Similarly, we find that

$$\begin{array}{ccc} E_{Z}\left[{\tilde{e}}_{2,h_{2}}^{3}\left(Z\right)\right] & = & \frac{1}{N_{2}^{2}h_{2}^{3d}}E_{Z}V_{i}^{3}\left(Z\right)\\ & = & \frac{1}{{\left(N_{2}h_{2}^{d}\right)}^{2}}\sum_{m=0}^{\left\lfloor s/2\right\rfloor }c_{3,2,3,m}\left(Z\right)h_{2}^{2m}+o\left(\frac{1}{N_{2}}\right). \end{array}$$

For q=4, we have

$$\begin{array}{ccc} E_{Z}\left[{\tilde{e}}_{2,h_{2}}^{4}\left(Z\right)\right] & = & \frac{1}{N_{2}^{3}h_{2}^{4d}}E_{Z}V_{i}^{4}\left(Z\right)+\frac{N_{2}-1}{N_{2}^{3}h_{2}^{4d}}{\left(E_{Z}V_{i}^{2}\left(Z\right)\right)}^{2}\\ & = & \frac{1}{{\left(N_{2}h_{2}^{d}\right)}^{3}}\sum_{m=0}^{\left\lfloor s/2\right\rfloor }c_{3,2,4,m}\left(Z\right)h_{2}^{2m}+\frac{1}{{\left(N_{2}h_{2}^{d}\right)}^{2}}\sum_{m=0}^{\left\lfloor s/2\right\rfloor }c_{6,2,2,m}\left(Z\right)h_{2}^{2m}+o\left(\frac{1}{N_{2}}\right). \end{array}$$

The pattern for q≥2 is then,

$$E_{Z}\left[{\tilde{e}}_{2,h_{2}}^{q}\left(Z\right)\right]=\sum_{i\in n(q)}\frac{1}{{\left(N_{2}h_{2}^{d}\right)}^{q-i}}\sum_{m=0}^{\left\lfloor s/2\right\rfloor }c_{6,2,q,i,m}\left(Z\right)h_{2}^{2m}+O\left(\frac{1}{N_{2}}\right).$$

For any integer (*q*), the largest possible factor is q/2. Thus, for a given *q*, the smallest possible exponent on the $N_{2}h_{2}^{d}$ term is q/2. This increases as *q* increases. A similar expression holds for $E_{Z}\left[{\tilde{e}}_{1,h_{1}}^{q}\left(Z\right)\right]$, except the $X_{i}$s are replaced with $Y_{i}$, $f_{2}$ is replaced with $f_{1}$, and $N_{2}$ and $h_{2}$ are replaced with $N_{1}$ and $h_{1}$, respectively, all resulting in different constants. Then, since ${\tilde{e}}_{1,h_{1}}\left(Z\right)$ and ${\tilde{e}}_{2,h_{2}}\left(Z\right)$ are conditionally independent given Z,

$$\begin{array}{ccc} E_{Z}\left[{\tilde{e}}_{1,h_{1}}^{q}\left(Z\right){\tilde{e}}_{2,h_{2}}^{l}\left(Z\right)\right] & = & \left(\sum_{i\in n(q)}\frac{1}{{\left(N_{1}h_{1}^{d}\right)}^{q-i}}\sum_{m=0}^{\left\lfloor s/2\right\rfloor }c_{6,1,q,i,m}\left(Z\right)h_{1}^{2m}\right)\left(\sum_{j\in n(l)}\frac{1}{{\left(N_{2}h_{2}^{d}\right)}^{l-j}}\sum_{t=0}^{\left\lfloor s/2\right\rfloor }c_{6,2,l,j,t}\left(Z\right)h_{2}^{2t}\right)\\ & & +O\left(\frac{1}{N_{1}}+\frac{1}{N_{2}}\right). \end{array}$$

 ☐

Applying Lemma A3 to (A18) when taking the conditional expectation given Z in the interior gives an expression of the form

(A21) $$\begin{array}{c} \sum_{j=1}^{\lambda /2}\sum_{m=0}^{\left\lfloor s/2\right\rfloor }\left(c_{7,1,j,m}\left(E_{Z}{\tilde{f}}_{1,h_{1}}\left(Z\right),E_{Z}{\tilde{f}}_{2,h_{2}}\left(Z\right)\right)\frac{h_{1}^{2m}}{{\left(N_{1}h_{1}^{d}\right)}^{j}}+c_{7,2,j,m}\left(E_{Z}{\tilde{f}}_{2,h_{2}}\left(Z\right),E_{Z}{\tilde{f}}_{2,h_{2}}\left(Z\right)\right)\frac{h_{2}^{2m}}{{\left(N_{2}h_{2}^{d}\right)}^{j}}\right)\\ +\sum_{j=1}^{\lambda /2}\sum_{m=0}^{\left\lfloor s/2\right\rfloor }\sum_{i=1}^{\lambda /2}\sum_{n=0}^{\left\lfloor s/2\right\rfloor }c_{7,j,i,m,n}\left(E_{Z}{\tilde{f}}_{2,h_{2}}\left(Z\right),E_{Z}{\tilde{f}}_{2,h_{2}}\left(Z\right)\right)\frac{h_{1}^{2m}h_{2}^{2n}}{{\left(N_{1}h_{1}^{d}\right)}^{j}{\left(N_{2}h_{2}^{d}\right)}^{i}}\\ +O\left(\frac{1}{{\left(N_{1}h_{1}^{d}\right)}^{\frac{\lambda }{2}}}+\frac{1}{{\left(N_{2}h_{2}^{d}\right)}^{\frac{\lambda }{2}}}\right). \end{array}$$

Note that the functionals $c_{7,i,j,m}$ and $c_{7,j,i,m,n}$ depend on the derivatives of *g* and $E_{Z}{\tilde{f}}_{i,h_{i}}\left(Z\right)$ which depend on $h_{i}$. To apply an ensemble estimation, we need to separate the dependence on $h_{i}$ from the constants. If we use ODin1, then it is sufficient to note that in the interior of the support, $E_{Z}{\tilde{f}}_{i,h_{i}}\left(Z\right)=f_{i}\left(Z\right)+o\left(1\right)$ and therefore, $c\left(E_{Z}{\tilde{f}}_{1,h_{1}}\left(Z\right),E_{Z}{\tilde{f}}_{2,h_{2}}\left(Z\right)\right)=c\left(f_{1}\left(Z\right),f_{2}\left(Z\right)\right)+o\left(1\right)$ for some functional *c*. The terms in (A22) reduce to

$$c_{7,1,1,0}\left(f_{1}\left(Z\right),f_{2}\left(Z\right)\right)\frac{1}{N_{1}h_{1}^{d}}+c_{7,2,1,0}\left(f_{1}\left(Z\right),f_{2}\left(Z\right)\right)\frac{1}{N_{2}h_{2}^{d}}+o\left(\frac{1}{N_{1}h_{1}^{d}}+\frac{1}{N_{2}h_{2}^{d}}\right).$$

For ODin2, we need the higher order terms. To separate the dependence on $h_{i}$ from the constants, we need more information about the functional *g* and its derivatives. Consider a special case where the functional g(x,y) has derivatives of the form of $x^{\alpha }y^{\beta }$ with α,β<0. This includes the important cases of the KL divergence and the Renyi divergence. The generalized binomial theorem states that if αm:=α(α−1)…(α−m+1)m! and if *q* and *t* are real numbers with |q|>|t|, then for any complex number (α),

(A22) (q+t)α=∑m=0∞αmqα−mtm.

Since the densities are bounded away from zero, for sufficiently small $h_{i}$, we have $f_{i}\left(Z\right)>\left|\sum_{j=\nu /2}^{\left\lfloor s/2\right\rfloor }c_{i,j}\left(Z\right)h_{i}^{2j}+O\left(h_{i}^{s}\right)\right|.$ Applying the generalized binomial theorem and Lemma A1 gives

EZf˜1,h1(Z)α=∑m=0∞αmfiα−m(Z)∑j=ν/2s/2ci,j(Z)hi2j+Ohism.

Since *m* is an integer, the exponents of the $h_{i}$ terms are also integers. Thus, (A22) gives, in this case,

(A23) $$\begin{array}{ccc} E_{Z}\left[g\left({\tilde{f}}_{1,h_{1}}\left(Z\right),{\tilde{f}}_{2,h_{2}}\left(Z\right)\right)-g\left(E_{Z}{\tilde{f}}_{1,h_{1}}\left(Z\right),E_{Z}{\tilde{f}}_{2,h_{2}}\left(Z\right)\right)\right] & = & \sum_{j=1}^{\lambda /2}\sum_{m=0}^{\left\lfloor s/2\right\rfloor }\left(c_{8,1,j,m}\left(Z\right)\frac{h_{1}^{2m}}{{\left(N_{1}h_{1}^{d}\right)}^{j}}+c_{8,2,j,m}\left(Z\right)\frac{h_{2}^{2m}}{{\left(N_{2}h_{2}^{d}\right)}^{j}}\right)\\ & & +\sum_{j=1}^{\lambda /2}\sum_{m=0}^{\left\lfloor s/2\right\rfloor }\sum_{i=1}^{\lambda /2}\sum_{n=0}^{\left\lfloor s/2\right\rfloor }c_{8,j,i,m,n}\left(Z\right)\frac{h_{1}^{2m}h_{2}^{2n}}{{\left(N_{1}h_{1}^{d}\right)}^{j}{\left(N_{2}h_{2}^{d}\right)}^{i}}\\ & & +O\left(\frac{1}{{\left(N_{1}h_{1}^{d}\right)}^{\frac{\lambda }{2}}}+\frac{1}{{\left(N_{2}h_{2}^{d}\right)}^{\frac{\lambda }{2}}}+h_{1}^{s}+h_{2}^{s}\right). \end{array}$$

As before, the case for Z close to the boundary of the support is more complicated. However, by using a similar technique to the proof of Lemma A2 for Z at the boundary and combining with previous results, we find that for general *g*,

(A24) $$E\left[g\left({\tilde{f}}_{1,h_{1}}\left(Z\right),{\tilde{f}}_{2,h_{2}}\left(Z\right)\right)-g\left(E_{Z}{\tilde{f}}_{1,h_{1}}\left(Z\right),E_{Z}{\tilde{f}}_{2,h_{2}}\left(Z\right)\right)\right]=c_{9,1}\frac{1}{N_{1}h_{1}^{d}}+c_{9,2}\frac{1}{N_{2}h_{2}^{d}}+o\left(\frac{1}{N_{1}h_{1}^{d}}+\frac{1}{N_{2}h_{2}^{d}}\right).$$

If g(x,y) has derivatives of the form of $x^{\alpha }y^{\beta }$ with α,β<0, then we can similarly obtain

(A25) $$\begin{array}{ccc} E\left[g\left({\tilde{f}}_{1,h_{1}}\left(Z\right),{\tilde{f}}_{2,h_{2}}\left(Z\right)\right)-g\left(E_{Z}{\tilde{f}}_{1,h_{1}}\left(Z\right),E_{Z}{\tilde{f}}_{2,h_{2}}\left(Z\right)\right)\right] & = & \sum_{j=1}^{\lambda /2}\sum_{m=0}^{r}\left(c_{9,1,j,m}\frac{h_{1}^{m}}{{\left(N_{1}h_{1}^{d}\right)}^{j}}+c_{9,2,j,m}\frac{h_{2}^{m}}{{\left(N_{2}h_{2}^{d}\right)}^{j}}\right)\\ & & +\sum_{j=1}^{\lambda /2}\sum_{m=0}^{r}\sum_{i=1}^{\lambda /2}\sum_{n=0}^{r}c_{9,j,i,m,n}\frac{h_{1}^{m}h_{2}^{n}}{{\left(N_{1}h_{1}^{d}\right)}^{j}{\left(N_{2}h_{2}^{d}\right)}^{i}}\\ & & +O\left(\frac{1}{{\left(N_{1}h_{1}^{d}\right)}^{\frac{\lambda }{2}}}+\frac{1}{{\left(N_{2}h_{2}^{d}\right)}^{\frac{\lambda }{2}}}+h_{1}^{s}+h_{2}^{s}\right). \end{array}$$

Combining (A17) with either (A24) or (A26) completes the proof.

## Appendix F. Proof of Theorem A4 (Variance)

To bound the variance of the plug-in estimator ${\tilde{G}}_{h_{1},h_{2}}$, we use the Efron–Stein inequality [70]:

**Lemma** **A4** (Efron–Stein Inequality)**.**
*Let $X_{1},\ldots ,X_{n},X_{1}^{{}^{\prime }},\ldots ,X_{n}^{{}^{\prime }}$ be independent random variables on the space S. Then, if f:S×…×S→R, we have*

$$V\left[f(X_{1},\ldots ,X_{n})\right]\leq \frac{1}{2}\sum_{i=1}^{n}E\left[{\left(f\left(X_{1},\ldots ,X_{n}\right)-f\left(X_{1},\ldots ,X_{i}^{{}^{\prime }},\ldots ,X_{n}\right)\right)}^{2}\right].$$

Suppose we have samples $\left\{X_{1},\ldots ,X_{N_{2}},Y_{1},\ldots ,Y_{N_{1}}\right\}$ and $\left\{X_{1}^{{}^{\prime }},\ldots ,X_{N_{2}},Y_{1},\ldots ,Y_{N_{1}}\right\}$ and denote the respective estimators as ${\tilde{G}}_{h_{1},h_{2}}$ and ${\tilde{G}}_{h_{1},h_{2}}^{{}^{\prime }}$. We have

(A26) $$\begin{array}{ccc} \left|{\tilde{G}}_{h_{1},h_{2}}-{\tilde{G}}_{h_{1},h_{2}}^{{}^{\prime }}\right| & \leq & \frac{1}{N_{2}}\left|g\left({\tilde{f}}_{1,h_{1}}\left(X_{1}\right),{\tilde{f}}_{2,h_{2}}\left(X_{1}\right)\right)-g\left({\tilde{f}}_{1,h_{1}}\left(X_{1}^{{}^{\prime }}\right),{\tilde{f}}_{2,h_{2}}\left(X_{1}^{{}^{\prime }}\right)\right)\right|\\ & & +\frac{1}{N_{2}}\sum_{j=2}^{N_{2}}\left|g\left({\tilde{f}}_{1,h_{1}}\left(X_{j}\right),{\tilde{f}}_{2,h_{2}}\left(X_{j}\right)\right)-g\left({\tilde{f}}_{1,h_{1}}\left(X_{j}\right),{\tilde{f}}_{2,h_{2}}^{{}^{\prime }}\left(X_{j}\right)\right)\right|. \end{array}$$

Since *g* is Lipschitz continuous with the constant $C_{g}$, we have

(A27) $$\begin{array}{ccc} \left|g\left({\tilde{f}}_{1,h_{1}}\left(X_{1}\right),{\tilde{f}}_{2,h_{2}}\left(X_{1}\right)\right)-g\left({\tilde{f}}_{1,h_{1}}\left(X_{1}^{{}^{\prime }}\right),{\tilde{f}}_{2,h_{2}}\left(X_{1}^{{}^{\prime }}\right)\right)\right| & \leq & C_{g}\left(\left|{\tilde{f}}_{1,h_{1}}\left(X_{1}\right)-{\tilde{f}}_{1,h_{1}}\left(X_{1}^{{}^{\prime }}\right)\right|+\left|{\tilde{f}}_{2,h_{2}}\left(X_{1}\right)-{\tilde{f}}_{2,h_{2}}\left(X_{1}^{{}^{\prime }}\right)\right|\right), \end{array}$$

(A28) $$\begin{array}{ccc} \left|{\tilde{f}}_{1,h_{1}}\left(X_{1}\right)-{\tilde{f}}_{1,h_{1}}\left(X_{1}^{{}^{\prime }}\right)\right| & = & \frac{1}{N_{1}h_{1}^{d}}\left|\sum_{i=1}^{N_{1}}\left(K\left(\frac{X_{1}-Y_{i}}{h_{1}}\right)-K\left(\frac{X_{1}^{{}^{\prime }}-Y_{i}}{h_{1}}\right)\right)\right|\\ & \leq & \frac{1}{N_{1}h_{1}^{d}}\sum_{i=1}^{N_{1}}\left|K\left(\frac{X_{1}-Y_{i}}{h_{1}}\right)-K\left(\frac{X_{1}^{{}^{\prime }}-Y_{i}}{h_{1}}\right)\right|\\ \Rightarrow E\left[{\left|{\tilde{f}}_{1,h_{1}}\left(X_{1}\right)-{\tilde{f}}_{1,h_{1}}\left(X_{1}^{{}^{\prime }}\right)\right|}^{2}\right] & \leq & \frac{1}{N_{1}h_{1}^{2d}}\sum_{i=1}^{N_{1}}E\left[{\left(K\left(\frac{X_{1}-Y_{i}}{h_{1}}\right)-K\left(\frac{X_{1}^{{}^{\prime }}-Y_{i}}{h_{1}}\right)\right)}^{2}\right], \end{array}$$

where the last step follows from Jensen’s inequality. By making the substitutions $u_{i}=\frac{X_{1}-Y_{i}}{h_{1}}$ and $u_{i}^{{}^{\prime }}=\frac{X_{1}^{{}^{\prime }}-Y_{i}}{h_{1}}$, this gives

$$\begin{array}{ccc} \frac{1}{h_{1}^{2d}}E\left[{\left(K\left(\frac{X_{1}-Y_{i}}{h_{1}}\right)-K\left(\frac{X_{1}^{{}^{\prime }}-Y_{i}}{h_{1}}\right)\right)}^{2}\right] & = & \frac{1}{h^{2d}}\int {\left(K\left(\frac{X_{1}-Y_{i}}{h_{1}}\right)-K\left(\frac{X_{1}^{{}^{\prime }}-Y_{i}}{h_{1}}\right)\right)}^{2}\times \\ & & f_{2}\left(X_{1}\right)f_{2}\left(X_{1}^{{}^{\prime }}\right)f_{1}\left(Y_{i}\right)dX_{1}dX_{1}^{{}^{\prime }}dY_{i}\\ & \leq & {2||K||}_{\infty }^{2}. \end{array}$$

Combining this with (A29) gives

$$E\left[{\left|{\tilde{f}}_{1,h_{1}}\left(X_{1}\right)-{\tilde{f}}_{1,h_{1}}\left(X_{1}^{{}^{\prime }}\right)\right|}^{2}\right]\leq {2||K||}_{\infty }^{2}.$$

Similarly,

$$E\left[{\left|{\tilde{f}}_{2,h_{2}}\left(X_{1}\right)-{\tilde{f}}_{2,h_{2}}\left(X_{1}^{{}^{\prime }}\right)\right|}^{2}\right]\leq {2||K||}_{\infty }^{2}.$$

Combining these results with (A27) gives

(A29) $$E\left[{\left(g\left({\tilde{f}}_{1,h_{1}}\left(X_{1}\right),{\tilde{f}}_{2,h_{2}}\left(X_{1}\right)\right)-g\left({\tilde{f}}_{1,h_{1}}\left(X_{1}^{{}^{\prime }}\right),{\tilde{f}}_{2,h_{2}}\left(X_{1}^{{}^{\prime }}\right)\right)\right)}^{2}\right]\leq 8C_{g}^{2}{\left||K|\right|}_{\infty }^{2}.$$

The second term in (A26) is controlled in a similar way. From the Lipschitz condition,

$$\begin{array}{ccc} {\left|g\left({\tilde{f}}_{1,h_{1}}\left(X_{j}\right),{\tilde{f}}_{2,h_{2}}\left(X_{j}\right)\right)-g\left({\tilde{f}}_{1,h_{1}}\left(X_{j}\right),{\tilde{f}}_{2,h_{2}}^{{}^{\prime }}\left(X_{j}\right)\right)\right|}^{2} & \leq & C_{g}^{2}{\left|{\tilde{f}}_{2,h_{2}}\left(X_{j}\right)-{\tilde{f}}_{2,h_{2}}^{{}^{\prime }}\left(X_{j}\right)\right|}^{2}\\ & = & \frac{C_{g}^{2}}{M_{2}^{2}h_{2}^{2d}}{\left(K\left(\frac{X_{j}-X_{1}}{h}\right)-K\left(\frac{X_{j}-X_{1}^{{}^{\prime }}}{h}\right)\right)}^{2}. \end{array}$$

The $h_{2}^{2d}$ terms are eliminated by making the substitutions of $u_{j}=\frac{X_{j}-X_{1}}{h_{2}}$ and $u_{j}^{{}^{\prime }}=\frac{X_{j}-X_{1}^{{}^{\prime }}}{h_{2}}$ within the expectation to obtain

(A30) $$\begin{array}{ccc} E\left[{\left|g\left({\tilde{f}}_{1,h_{1}}\left(X_{j}\right),{\tilde{f}}_{2,h_{2}}\left(X_{j}\right)\right)-g\left({\tilde{f}}_{1,h_{1}}\left(X_{j}\right),{\tilde{f}}_{2,h_{2}}^{{}^{\prime }}\left(X_{j}\right)\right)\right|}^{2}\right] & \leq & \frac{2C_{g}^{2}{\left||K|\right|}_{\infty }^{2}}{M_{2}^{2}} \end{array}$$

$$\Rightarrow E\left[{\left(\sum_{j=2}^{N_{2}}\left|g\left({\tilde{f}}_{1,h_{1}}\left(X_{j}\right),{\tilde{f}}_{2,h_{2}}\left(X_{j}\right)\right)-g\left({\tilde{f}}_{1,h_{1}}\left(X_{j}\right),{\tilde{f}}_{2,h_{2}}^{{}^{\prime }}\left(X_{j}\right)\right)\right|\right)}^{2}\right]$$

(A31) $$\begin{array}{cc} = & \sum_{j=2}^{N_{2}}\sum_{i=2}^{N_{2}}E\left[\left|g\left({\tilde{f}}_{1,h_{1}}\left(X_{j}\right),{\tilde{f}}_{2,h_{2}}\left(X_{j}\right)\right)-g\left({\tilde{f}}_{1,h_{1}}\left(X_{j}\right),{\tilde{f}}_{2,h_{2}}^{{}^{\prime }}\left(X_{j}\right)\right)\right|\left|g\left({\tilde{f}}_{1,h_{1}}\left(X_{i}\right),{\tilde{f}}_{2,h_{2}}\left(X_{i}\right)\right)-g\left({\tilde{f}}_{1,h_{1}}\left(X_{i}\right),{\tilde{f}}_{2,h_{2}}^{{}^{\prime }}\left(X_{i}\right)\right)\right|\right]\\ \leq & M_{2}^{2}E\left[{\left|g\left({\tilde{f}}_{1,h_{1}}\left(X_{j}\right),{\tilde{f}}_{2,h_{2}}\left(X_{j}\right)\right)-g\left({\tilde{f}}_{1,h_{1}}\left(X_{j}\right),{\tilde{f}}_{2,h_{2}}^{{}^{\prime }}\left(X_{j}\right)\right)\right|}^{2}\right]\\ \leq & 2C_{g}^{2}{\left||K|\right|}_{\infty }^{2}, \end{array}$$

where we use the Cauchy Schwarz inequality to bound the expectation within each summand. Finally, applying Jensen’s inequality and (A29) and (A32) gives

$$\begin{array}{ccc} E\left[{\left|{\tilde{G}}_{h_{1},h_{2}}-{\tilde{G}}_{h_{1},h_{2}}^{{}^{\prime }}\right|}^{2}\right] & \leq & \frac{2}{N_{2}^{2}}E\left[{\left|g\left({\tilde{f}}_{1,h_{1}}\left(X_{1}\right),{\tilde{f}}_{2,h_{2}}\left(X_{1}\right)\right)-g\left({\tilde{f}}_{1,h_{1}}\left(X_{1}^{{}^{\prime }}\right),{\tilde{f}}_{2,h_{2}}\left(X_{1}^{{}^{\prime }}\right)\right)\right|}^{2}\right]\\ & & +\frac{2}{N_{2}^{2}}E\left[{\left(\sum_{j=2}^{N_{2}}\left|g\left({\tilde{f}}_{1,h_{1}}\left(X_{j}\right),{\tilde{f}}_{2,h_{2}}\left(X_{j}\right)\right)-g\left({\tilde{f}}_{1,h_{1}}\left(X_{j}\right),{\tilde{f}}_{2,h_{2}}^{{}^{\prime }}\left(X_{j}\right)\right)\right|\right)}^{2}\right]\\ & \leq & \frac{20C_{g}^{2}{\left||K|\right|}_{\infty }^{2}}{N_{2}^{2}}. \end{array}$$

Now, suppose we have samples $\left\{X_{1},\ldots ,X_{N_{2}},Y_{1},\ldots ,Y_{N_{1}}\right\}$ and $\left\{X_{1},\ldots ,X_{N_{2}},Y_{1}^{{}^{\prime }},\ldots ,Y_{N_{1}}\right\}$ and denote the respective estimators as ${\tilde{G}}_{h_{1},h_{2}}$ and ${\tilde{G}}_{h_{1},h_{2}}^{{}^{\prime }}$. Then,

$$\begin{array}{ccc} \left|g\left({\tilde{f}}_{1,h_{1}}\left(X_{j}\right),{\tilde{f}}_{2,h_{2}}\left(X_{j}\right)\right)-g\left({\tilde{f}}_{1,h_{1}}^{{}^{\prime }}\left(X_{j}\right),{\tilde{f}}_{2,h_{2}}\left(X_{j}\right)\right)\right| & \leq & C_{g}\left|{\tilde{f}}_{1,h_{1}}\left(X_{j}\right)-{\tilde{f}}_{1,h_{1}}^{{}^{\prime }}\left(X_{j}\right)\right|\\ & = & \frac{C_{g}}{N_{1}h_{1}^{d}}\left|K\left(\frac{X_{j}-Y_{1}}{h_{1}}\right)-K\left(\frac{X_{j}-Y_{1}^{{}^{\prime }}}{h_{1}}\right)\right|\\ \Rightarrow E\left[{\left|g\left({\tilde{f}}_{1,h_{1}}\left(X_{j}\right),{\tilde{f}}_{2,h_{2}}\left(X_{j}\right)\right)-g\left({\tilde{f}}_{1,h_{1}}^{{}^{\prime }}\left(X_{j}\right),{\tilde{f}}_{2,h_{2}}\left(X_{j}\right)\right)\right|}^{2}\right] & \leq & \frac{2C_{g}^{2}{\left||K|\right|}_{\infty }^{2}}{N_{1}^{2}}. \end{array}$$

Thus, using a similar argument as was used to obtain (A32),

$$\begin{array}{ccc} E\left[{\left|{\tilde{G}}_{h_{1},h_{2}}-{\tilde{G}}_{h_{1},h_{2}}^{{}^{\prime }}\right|}^{2}\right] & \leq & \frac{1}{N_{2}^{2}}E\left[{\left(\sum_{j=1}^{N_{2}}\left|g\left({\tilde{f}}_{1,h_{1}}\left(X_{j}\right),{\tilde{f}}_{2,h_{2}}\left(X_{j}\right)\right)-g\left({\tilde{f}}_{1,h_{1}}^{{}^{\prime }}\left(X_{j}\right),{\tilde{f}}_{2,h_{2}}\left(X_{j}\right)\right)\right|\right)}^{2}\right]\\ & \leq & \frac{2C_{g}^{2}{\left||K|\right|}_{\infty }^{2}}{N_{2}^{2}}. \end{array}$$

Applying the Efron–Stein inequality gives

$$V\left[{\tilde{G}}_{h_{1},h_{2}}\right]\leq \frac{10C_{g}^{2}{\left||K|\right|}_{\infty }^{2}}{N_{2}}+\frac{C_{g}^{2}{\left||K|\right|}_{\infty }^{2}N_{1}}{N_{2}^{2}}.$$

## Appendix G. Proof of Theorem 4 (CLT)

We are interested in the asymptotic distribution of

$$\begin{array}{ccc} \sqrt{N_{2}}\left({\tilde{G}}_{h_{1},h_{2}}-E\left[{\tilde{G}}_{h_{1},h_{2}}\right]\right) & = & \frac{1}{\sqrt{N_{2}}}\sum_{j=1}^{N_{2}}\left(g\left({\tilde{f}}_{1,h_{1}}\left(X_{j}\right),{\tilde{f}}_{2,h_{2}}\left(X_{j}\right)\right)-E_{X_{j}}\left[g\left({\tilde{f}}_{1,h_{1}}\left(X_{j}\right),{\tilde{f}}_{2,h_{2}}\left(X_{j}\right)\right)\right]\right)\\ & & +\frac{1}{\sqrt{N_{2}}}\sum_{j=1}^{N_{2}}\left(E_{X_{j}}\left[g\left({\tilde{f}}_{1,h_{1}}\left(X_{j}\right),{\tilde{f}}_{2,h_{2}}\left(X_{j}\right)\right)\right]-E\left[g\left({\tilde{f}}_{1,h_{1}}\left(X_{j}\right),{\tilde{f}}_{2,h_{2}}\left(X_{j}\right)\right)\right]\right). \end{array}$$

Note that in the standard central limit theorem [79], the second term converges in distribution to a Gaussian random variable. If the first term converges in probability to a constant (specifically, 0), then we can use Slutsky’s theorem [80] to find the asymptotic distribution. So, now, we focus on the first term which we denote as $V_{N_{2}}$.

To prove convergence in probability, we use Chebyshev’s inequality. Note that $E\left[V_{N_{2}}\right]=0$. To bound the variance of $V_{N_{2}}$, we again use the Efron–Stein inequality. Let $X_{1}^{{}^{\prime }}$ be drawn from $f_{2}$ and denote $V_{N_{2}}$ and $V_{N_{2}}^{{}^{\prime }}$ as the sequences using $X_{1}$ and $X_{1}^{{}^{\prime }}$, respectively. Then,

(A32) $$\begin{array}{ccc} V_{N_{2}}-V_{N_{2}}^{{}^{\prime }} & = & \frac{1}{\sqrt{N_{2}}}\left(g\left({\tilde{f}}_{1,h_{1}}\left(X_{1}\right),{\tilde{f}}_{2,h_{2}}\left(X_{1}\right)\right)-E_{X_{1}}\left[g\left({\tilde{f}}_{1,h_{1}}\left(X_{1}\right),{\tilde{f}}_{2,h_{2}}\left(X_{1}\right)\right)\right]\right)\\ & & -\frac{1}{\sqrt{N_{2}}}\left(g\left({\tilde{f}}_{1,h_{1}}\left(X_{1}^{{}^{\prime }}\right),{\tilde{f}}_{2,h_{2}}\left(X_{1}^{{}^{\prime }}\right)\right)-E_{X_{1}^{{}^{\prime }}}\left[g\left({\tilde{f}}_{1,h_{1}}\left(X_{1}^{{}^{\prime }}\right),{\tilde{f}}_{2,h_{2}}\left(X_{1}^{{}^{\prime }}\right)\right)\right]\right)\\ & & +\frac{1}{\sqrt{N_{2}}}\sum_{j=2}^{N_{2}}\left(g\left({\tilde{f}}_{1,h_{1}}\left(X_{j}\right),{\tilde{f}}_{2,h_{2}}\left(X_{j}\right)\right)-g\left({\tilde{f}}_{1,h_{1}}\left(X_{j}\right),{\tilde{f}}_{2,h_{2}}^{{}^{\prime }}\left(X_{j}\right)\right)\right). \end{array}$$

Note that

$$E\left[{\left(g\left({\tilde{f}}_{1,h_{1}}\left(X_{1}\right),{\tilde{f}}_{2,h_{2}}\left(X_{1}\right)\right)-E_{X_{1}}\left[g\left({\tilde{f}}_{1,h_{1}}\left(X_{1}\right),{\tilde{f}}_{2,h_{2}}\left(X_{1}\right)\right)\right]\right)}^{2}\right]=E\left[V_{X_{1}}\left[g\left({\tilde{f}}_{1,h_{1}}\left(X_{1}\right),{\tilde{f}}_{2,h_{2}}\left(X_{1}\right)\right)\right]\right].$$

We use the Efron–Stein inequality to bound $V_{X_{1}}\left[g\left({\tilde{f}}_{1,h_{1}}\left(X_{1}\right),{\tilde{f}}_{2,h_{2}}\left(X_{1}\right)\right)\right]$.

We do this by bounding the conditional expectation of the term

$$\left|g\left({\tilde{f}}_{1,h_{1}}\left(X_{1}\right),{\tilde{f}}_{2,h_{2}}\left(X_{1}\right)\right)-g\left({\tilde{f}}_{1,h_{1}}\left(X_{1}\right),{\tilde{f}}_{2,h_{2}}^{{}^{\prime }}\left(X_{1}\right)\right)\right|,$$

where $X_{i}$ is replaced with $X_{i}^{{}^{\prime }}$ in the KDEs for some i≠1. Using similar steps as in Appendix F, we have

$$E_{X_{1}}\left[{\left|g\left({\tilde{f}}_{1,h_{1}}\left(X_{1}\right),{\tilde{f}}_{2,h_{2}}\left(X_{1}\right)\right)-g\left({\tilde{f}}_{1,h_{1}}\left(X_{1}\right),{\tilde{f}}_{2,h_{2}}^{{}^{\prime }}\left(X_{1}\right)\right)\right|}^{2}\right]=O\left(\frac{1}{N_{2}^{2}}\right).$$

A similar result is obtained when $Y_{i}$ is replaced with $Y_{i}^{{}^{\prime }}$. Then, based on the Efron–Stein inequality, $V_{X_{1}}\left[g\left({\tilde{f}}_{1,h_{1}}\left(X_{1}\right),{\tilde{f}}_{2,h_{2}}\left(X_{1}\right)\right)\right]=O\left(\frac{1}{N_{2}}+\frac{1}{N_{1}}\right)$.

Therefore,

$$E\left[\frac{1}{N_{2}}{\left(g\left({\tilde{f}}_{1,h_{1}}\left(X_{1}\right),{\tilde{f}}_{2,h_{2}}\left(X_{1}\right)\right)-E_{X_{1}}\left[g\left({\tilde{f}}_{1,h_{1}}\left(X_{1}\right),{\tilde{f}}_{2,h_{2}}\left(X_{1}\right)\right)\right]\right)}^{2}\right]=O\left(\frac{1}{N_{2}^{2}}+\frac{1}{N_{1}N_{2}}\right).$$

A similar result holds for the $g\left({\tilde{f}}_{1,h_{1}}\left(X_{1}^{{}^{\prime }}\right),{\tilde{f}}_{2,h_{2}}\left(X_{1}^{{}^{\prime }}\right)\right)$ terms in (A33).

For the third term in (A33),

$$E\left[{\left(\sum_{j=2}^{N_{2}}\left|g\left({\tilde{f}}_{1,h_{1}}\left(X_{j}\right),{\tilde{f}}_{2,h_{2}}\left(X_{j}\right)\right)-g\left({\tilde{f}}_{1,h_{1}}\left(X_{j}\right),{\tilde{f}}_{2,h_{2}}^{{}^{\prime }}\left(X_{j}\right)\right)\right|\right)}^{2}\right]$$

$$=\sum_{j=2}^{N_{2}}\sum_{i=2}^{N_{2}}E\left[\left|g\left({\tilde{f}}_{1,h_{1}}\left(X_{j}\right),{\tilde{f}}_{2,h_{2}}\left(X_{j}\right)\right)-g\left({\tilde{f}}_{1,h_{1}}\left(X_{j}\right),{\tilde{f}}_{2,h_{2}}^{{}^{\prime }}\left(X_{j}\right)\right)\right|\left|g\left({\tilde{f}}_{1,h_{1}}\left(X_{i}\right),{\tilde{f}}_{2,h_{2}}\left(X_{i}\right)\right)-g\left({\tilde{f}}_{1,h_{1}}\left(X_{i}\right),{\tilde{f}}_{2,h_{2}}^{{}^{\prime }}\left(X_{i}\right)\right)\right|\right].$$

There are $M_{2}$ terms where i=j, and we have from Appendix F (see (A30)) that

$$E\left[{\left|g\left({\tilde{f}}_{1,h_{1}}\left(X_{j}\right),{\tilde{f}}_{2,h_{2}}\left(X_{j}\right)\right)-g\left({\tilde{f}}_{1,h_{1}}\left(X_{j}\right),{\tilde{f}}_{2,h_{2}}^{{}^{\prime }}\left(X_{j}\right)\right)\right|}^{2}\right]\leq \frac{2C_{g}^{2}{\left||K|\right|}_{\infty }^{2}}{M_{2}^{2}}.$$

Thus, these terms are $O\left(\frac{1}{M_{2}}\right)$. There are $M_{2}^{2}-M_{2}$ terms when i≠j. In this case, we can do four substitutions of the form $u_{j}=\frac{X_{j}-X_{1}}{h_{2}}$ to obtain

$$E\left[\left|g\left({\tilde{f}}_{1,h_{1}}\left(X_{j}\right),{\tilde{f}}_{2,h_{2}}\left(X_{j}\right)\right)-g\left({\tilde{f}}_{1,h_{1}}\left(X_{j}\right),{\tilde{f}}_{2,h_{2}}^{{}^{\prime }}\left(X_{j}\right)\right)\right|\left|g\left({\tilde{f}}_{1,h_{1}}\left(X_{i}\right),{\tilde{f}}_{2,h_{2}}\left(X_{i}\right)\right)-g\left({\tilde{f}}_{1,h_{1}}\left(X_{i}\right),{\tilde{f}}_{2,h_{2}}^{{}^{\prime }}\left(X_{i}\right)\right)\right|\right]\leq \frac{4C_{g}^{2}{\left||K|\right|}_{\infty }^{2}h_{2}^{2d}}{M_{2}^{2}}.$$

Then, since $h_{2}^{d}=o\left(1\right)$, we get

(A33) $$E\left[{\left(\sum_{j=2}^{N_{2}}\left|g\left({\tilde{f}}_{1,h_{1}}\left(X_{j}\right),{\tilde{f}}_{2,h_{2}}\left(X_{j}\right)\right)-g\left({\tilde{f}}_{1,h_{1}}\left(X_{j}\right),{\tilde{f}}_{2,h_{2}}^{{}^{\prime }}\left(X_{j}\right)\right)\right|\right)}^{2}\right]=o\left(1\right),$$

$$\begin{array}{ccc} \Rightarrow E\left[{\left(V_{N_{2}}-V_{N_{2}}^{{}^{\prime }}\right)}^{2}\right] & \leq & \frac{3}{N_{2}}E\left[{\left(g\left({\tilde{f}}_{1,h_{1}}\left(X_{1}\right),{\tilde{f}}_{2,h_{2}}\left(X_{1}\right)\right)-E_{X_{1}}\left[g\left({\tilde{f}}_{1,h_{1}}\left(X_{1}\right),{\tilde{f}}_{2,h_{2}}\left(X_{1}\right)\right)\right]\right)}^{2}\right]\\ & & +\frac{3}{N_{2}}E\left[{\left(g\left({\tilde{f}}_{1,h_{1}}\left(X_{1}^{{}^{\prime }}\right),{\tilde{f}}_{2,h_{2}}\left(X_{1}^{{}^{\prime }}\right)\right)-E_{X_{1}^{{}^{\prime }}}\left[g\left({\tilde{f}}_{1,h_{1}}\left(X_{1}^{{}^{\prime }}\right),{\tilde{f}}_{2,h_{2}}\left(X_{1}^{{}^{\prime }}\right)\right)\right]\right)}^{2}\right]\\ & & +\frac{3}{N_{2}}E\left[{\left(\sum_{j=2}^{N_{2}}\left(g\left({\tilde{f}}_{1,h_{1}}\left(X_{j}\right),{\tilde{f}}_{2,h_{2}}\left(X_{j}\right)\right)-g\left({\tilde{f}}_{1,h_{1}}^{{}^{\prime }}\left(X_{j}\right),{\tilde{f}}_{2,h_{2}}^{{}^{\prime }}\left(X_{j}\right)\right)\right)\right)}^{2}\right]\\ & = & o\left(\frac{1}{N_{2}}\right). \end{array}$$

Now, consider samples $\left\{X_{1},\ldots ,X_{N_{2}},Y_{1},\ldots ,Y_{N_{1}}\right\}$ and $\left\{X_{1},\ldots ,X_{N_{2}},Y_{1}^{{}^{\prime }},\ldots ,Y_{N_{1}}\right\}$ and the respective sequences $V_{N_{2}}$ and $V_{N_{2}}^{{}^{\prime }}$. Then,

$$\begin{array}{ccc} V_{N_{2}}-V_{N_{2}}^{{}^{\prime }} & = & \frac{1}{\sqrt{N_{2}}}\sum_{j=1}^{N_{2}}\left(g\left({\tilde{f}}_{1,h_{1}}\left(X_{j}\right),{\tilde{f}}_{2,h_{2}}\left(X_{j}\right)\right)-g\left({\tilde{f}}_{1,h_{1}}^{{}^{\prime }}\left(X_{j}\right),{\tilde{f}}_{2,h_{2}}\left(X_{j}\right)\right)\right). \end{array}$$

Using a similar argument as that used to obtain (A33), we have that if $h_{1}^{d}=o\left(1\right)$ and $N_{1}\to \infty$, then

$$E\left[{\left(\sum_{j=2}^{N_{2}}\left|g\left({\tilde{f}}_{1,h_{1}}\left(X_{j}\right),{\tilde{f}}_{2,h_{2}}\left(X_{j}\right)\right)-g\left({\tilde{f}}_{1,h_{1}}^{{}^{\prime }}\left(X_{j}\right),{\tilde{f}}_{2,h_{2}}\left(X_{j}\right)\right)\right|\right)}^{2}\right]=o\left(1\right)$$

$$\Rightarrow E\left[{\left(V_{N_{2}}-V_{N_{2}}^{{}^{\prime }}\right)}^{2}\right]=o\left(\frac{1}{N_{2}}\right).$$

Applying the Efron–Stein inequality gives

$$V\left[V_{N_{2}}\right]=o\left(\frac{N_{2}+N_{1}}{N_{2}}\right)=o\left(1\right).$$

Thus, based on Chebyshev’s inequality,

$$\mathrm{Pr}\left(\left|V_{N_{2}}\right|>\epsilon \right)\leq \frac{V\left[V_{N_{2}}\right]}{\epsilon^{2}}=o\left(1\right),$$

and therefore, $V_{N_{2}}$ converges to zero in probability. Based on Slutsky’s theorem, $\sqrt{N_{2}}\left({\tilde{G}}_{h_{1},h_{2}}-E\left[{\tilde{G}}_{h_{1},h_{2}}\right]\right)$ converges in distribution to a zero mean Gaussian random variable with variance

$$V\left[E_{X}\left[g\left({\tilde{f}}_{1,h_{1}}\left(X\right),{\tilde{f}}_{2,h_{2}}\left(X\right)\right)\right]\right],$$

where X is drawn from $f_{2}$.

For the weighted ensemble estimator, we wish to know the asymptotic distribution of $\sqrt{N_{2}}\left({\tilde{G}}_{w}-E\left[{\tilde{G}}_{w}\right]\right)$ where ${\tilde{G}}_{w}=\sum_{l\in \bar{l}}w\left(l\right){\tilde{G}}_{h(l)}$. We have

$$\begin{array}{ccc} \sqrt{N_{2}}\left({\tilde{G}}_{w}-E\left[{\tilde{G}}_{w}\right]\right) & = & \frac{1}{\sqrt{N_{2}}}\sum_{j=1}^{N_{2}}\sum_{l\in \bar{l}}w\left(l\right)\left(g\left({\tilde{f}}_{1,h(l)}\left(X_{j}\right),{\tilde{f}}_{2,h(l)}\left(X_{j}\right)\right)-E_{X_{j}}\left[g\left({\tilde{f}}_{1,h(l)}\left(X_{j}\right),{\tilde{f}}_{2,h(l)}\left(X_{j}\right)\right)\right]\right)\\ & & +\frac{1}{\sqrt{N_{2}}}\sum_{j=1}^{N_{2}}\left(E_{X_{j}}\left[\sum_{l\in \bar{l}}w\left(l\right)g\left({\tilde{f}}_{1,h(l)}\left(X_{j}\right),{\tilde{f}}_{2,h(l)}\left(X_{j}\right)\right)\right]-E\left[\sum_{l\in \bar{l}}w\left(l\right)g\left({\tilde{f}}_{1,h(l)}\left(X_{j}\right),{\tilde{f}}_{2,h(l)}\left(X_{j}\right)\right)\right]\right). \end{array}$$

The second term again converges in distribution to a Gaussian random variable by the central limit theorem. The mean and variance are, respectively, zero and

$$V\left[\sum_{l\in \bar{l}}w\left(l\right)E_{X}\left[g\left({\tilde{f}}_{1,h(l)}\left(X\right),{\tilde{f}}_{2,h(l)}\left(X\right)\right)\right]\right].$$

The first term is equal to

$$\begin{array}{ccc} \sum_{l\in \bar{l}}w\left(l\right)\left(\frac{1}{\sqrt{N_{2}}}\sum_{j=1}^{N_{2}}\left(g\left({\tilde{f}}_{1,h(l)}\left(X_{j}\right),{\tilde{f}}_{2,h(l)}\left(X_{j}\right)\right)-E_{X_{j}}\left[g\left({\tilde{f}}_{1,h(l)}\left(X_{j}\right),{\tilde{f}}_{2,h(l)}\left(X_{j}\right)\right)\right]\right)\right) & = & \sum_{l\in \bar{l}}w\left(l\right)o_{P}\left(1\right)\\ & = & o_{P}\left(1\right), \end{array}$$

where $o_{P}\left(1\right)$ denotes the convergence to zero as a probability. In the last step, we use the fact that if two random variables converge in probability to constants, then their linear combination converges in probability to the linear combination of the constants. Combining this result with Slutsky’s theorem completes the proof.

## Appendix H. Proof of Theorem 5 (Uniform MSE)

Since the MSE is equal to the square of the bias plus the variance, we can upper bound the left hand side of (7) with

$$\begin{array}{cc} \underset{p,q\in \Sigma (s,K_{H},\epsilon_{0},\epsilon_{\infty })}{\sup}E\left[{\left({\tilde{G}}_{w_{0}}-G\left(p,q\right)\right)}^{2}\right] & =\underset{p,q\in \Sigma (s,K_{H},\epsilon_{0},\epsilon_{\infty })}{\sup}\left(\mathrm{Bias}{\left({\tilde{G}}_{w_{0}}\right)}^{2}+\mathrm{Var}\left({\tilde{G}}_{w_{0}}\right)\right)\\ & \leq \underset{p,q\in \Sigma (s,K_{H},\epsilon_{0},\epsilon_{\infty })}{\sup}\mathrm{Bias}{\left({\tilde{G}}_{w_{0}}\right)}^{2}+\underset{p,q\in \Sigma (s,K_{H},\epsilon_{0},\epsilon_{\infty })}{\sup}\mathrm{Var}\left({\tilde{G}}_{w_{0}}\right). \end{array}$$

From the assumptions (Lipschitz, kernel bounded, weight calculated from the relaxed optimization problem), we have

$$\begin{array}{ccc} \underset{p,q\in \Sigma (s,K_{H},\epsilon_{0},\epsilon_{\infty })}{\sup}\mathrm{Var}\left({\tilde{G}}_{w_{0}}\right) & \leq & \underset{p,q\in \Sigma (s,K,\epsilon_{0},\epsilon_{\infty })}{\sup}\frac{11C_{g}^{2}\left|\right|w_{0}{\left|\right|}_{2}^{2}{\left||K|\right|}_{\infty }}{N}\\ & = & \frac{11C_{g}^{2}\left|\right|w_{0}{\left|\right|}_{2}^{2}{\left||K|\right|}_{\infty }}{N}, \end{array}$$

where the last step follows on from the fact that all of the terms are independent of *p* and *q*.

For the bias, recall that if *g* is infinitely differentiable and if the optimal weight ($w_{0}$) is calculated using the relaxed convex optimization problem, then

(A34) $$\begin{array}{ccc} \mathrm{Bias}\left({\tilde{G}}_{w_{0}}\right) & = & \sum_{i\in J}c_{i}\left(p,q\right)\epsilon N^{-1/2},\\ \Rightarrow \mathrm{Bias}{\left({\tilde{G}}_{w_{0}}\right)}^{2} & = & \frac{\epsilon^{2}}{N}{\left(\sum_{i\in J}c_{i}\left(p,q\right)\right)}^{2}. \end{array}$$

We use a topology argument to bound the supremum of this term. We use the Extreme Value Theorem [81]:

**Theorem** **A5** **(Extreme** **Value** **Theorem)**
*Let f:X→R be continuous. If X is compact, then points c,d∈X s.t. f(c)≤f(x)≤f(d) exist for every x∈X.*

Based on this theorem, *f* achieves its minimum and maximum on *X*. Our approach is to first show that the functionals $c_{i}\left(p,q\right)$ are continuous wrt *p* and *q* in some appropriate norm. We then show that the space $\Sigma (s,K_{H},\epsilon_{0},\epsilon_{\infty })$ is compact wrt this norm. The Extreme Value Theorem can then be applied to bound the supremum of (A34).

We first define the norm. Let α=s−r>0. We use the standard norm on the space $\Sigma (s,K_{H})$ [82]:

$$\begin{array}{ccc} \left||f|\right| & = & {\left||f|\right|}_{\Sigma (s,K_{H})}\\ & = & {\left||f|\right|}_{C^{r}}+\max_{|\beta |=r}{\left|D^{\beta }f\right|}_{C^{0,\alpha }} \end{array}$$

where

$$\begin{array}{ccc} {\left||f|\right|}_{C^{r}} & = & \max_{|\beta |\leq r}\underset{x\in S}{\sup}\left|D^{\beta }f\left(x\right)\right|,\\ {\left|f\right|}_{C^{0,\alpha }} & = & \underset{x\neq y\in S}{\sup}\frac{\left|f(x)-f(y)\right|}{{\left|x-y\right|}^{\alpha }}. \end{array}$$

**Lemma** **A5.**
*The functionals $c_{i}\left(p,q\right)$ are continuous wrt the norm $\max(||p|{|}_{C^{r}},||q|{|}_{C^{r}})$.*

**Proof.** The functionals $c_{i}\left(p,q\right)$ depend on terms of the form

(A35) $$c\left(p,q\right)=\int \left({\left.\frac{\partial^{i+j}g\left(t_{1},t_{2}\right)}{\partial t_{1}^{i}\partial t_{2}^{j}}\right|}_{\begin{array}{c} t_{1}=p\left(x\right)\\ t_{2}=q\left(x\right) \end{array}}\right)D^{\beta }p\left(x\right)D^{\gamma }q\left(x\right)q\left(x\right)dx.$$

It is sufficient to show that this is continuous. Let ϵ>0 and $\max\left(||p-p_{0}{\left|\right|}_{C^{r}},||q-q_{0}{\left|\right|}_{C^{r}}\right)<\delta$ where δ>0 will be chosen later. Then, by applying the triangle inequality for integration and adding and subtracting terms, we have

$$|c\left(p,q\right)-c\left(p_{0},q_{0}\right)|$$

(A36) $$\begin{array}{cc} \leq & \int \left|\left({\left.\frac{\partial^{i+j}g\left(t_{1},t_{2}\right)}{\partial t_{1}^{i}\partial t_{2}^{j}}\right|}_{\begin{array}{c} t_{1}=p\left(x\right)\\ t_{2}=q\left(x\right) \end{array}}\right)D^{\beta }p\left(x\right)D^{\gamma }q\left(x\right)\left(q\left(x\right)-q_{0}\left(x\right)\right)\right|dx\\ & +\int \left|\left({\left.\frac{\partial^{i+j}g\left(t_{1},t_{2}\right)}{\partial t_{1}^{i}\partial t_{2}^{j}}\right|}_{\begin{array}{c} t_{1}=p\left(x\right)\\ t_{2}=q\left(x\right) \end{array}}\right)D^{\beta }p\left(x\right)q_{0}\left(x\right)\left(D^{\gamma }q\left(x\right)-D^{\gamma }q_{0}\left(x\right)\right)\right|dx\\ & +\int \left|D^{\beta }p_{0}\left(x\right)D^{\gamma }q_{0}\left(x\right)q_{0}\left(x\right)\left(\left({\left.\frac{\partial^{i+j}g\left(t_{1},t_{2}\right)}{\partial t_{1}^{i}\partial t_{2}^{j}}\right|}_{\begin{array}{c} t_{1}=p\left(x\right)\\ t_{2}=q\left(x\right) \end{array}}\right)\right.\right.\\ & -\left.\left.\left({\left.\frac{\partial^{i+j}g\left(t_{1},t_{2}\right)}{\partial t_{1}^{i}\partial t_{2}^{j}}\right|}_{\begin{array}{c} t_{1}=p_{0}\left(x\right)\\ t_{2}=q_{0}\left(x\right) \end{array}}\right)\right)\right|dx\\ & +\int \left|\left({\left.\frac{\partial^{i+j}g\left(t_{1},t_{2}\right)}{\partial t_{1}^{i}\partial t_{2}^{j}}\right|}_{\begin{array}{c} t_{1}=p\left(x\right)\\ t_{2}=q\left(x\right) \end{array}}\right)D^{\gamma }q_{0}\left(x\right)q_{0}\left(x\right)\left(D^{\beta }p\left(x\right)-D^{\beta }p_{0}\left(x\right)\right)\right|dx. \end{array}$$

Based on Assumption A.4, the absolute value of the mixed derivatives of *g* is bounded on the range defined for *p* and *q* by some constant ($C_{i,j}$). Also, $q_{0}\left(x\right)\leq \epsilon_{\infty }$. Furthermore, since $D^{\gamma }q_{0}$ and $D^{\beta }p$ are continuous, and since $S\subset R^{d}$ is compact, then the absolute value of derivatives $D^{\gamma }q_{0}$ and $D^{\beta }p$ is also bounded by a constant ($\epsilon_{\infty }^{{}^{\prime }}$). Let $\delta_{0}>0$. Then, since the mixed derivatives of *g* are continuous on the interval $\left[\epsilon_{0},\epsilon_{\infty }\right]$, they are uniformly continuous. Therefore, we can choose a small enough δ such that (s.t.)

(A37) $$\left|\left({\left.\frac{\partial^{i+j}g\left(t_{1},t_{2}\right)}{\partial t_{1}^{i}\partial t_{2}^{j}}\right|}_{\begin{array}{c} t_{1}=p\left(x\right)\\ t_{2}=q\left(x\right) \end{array}}\right)-\left({\left.\frac{\partial^{i+j}g\left(t_{1},t_{2}\right)}{\partial t_{1}^{i}\partial t_{2}^{j}}\right|}_{\begin{array}{c} t_{1}=p_{0}\left(x\right)\\ t_{2}=q_{0}\left(x\right) \end{array}}\right)\right|<\delta_{0}.$$

Combining all of these results with (A36) gives

$$\begin{array}{ccc} |c\left(p,q\right)-c\left(p_{0},q_{0}\right)| & \leq & \lambda \left(S\right)\delta C_{ij}\epsilon_{\infty }^{{}^{\prime }}\left(2+\epsilon_{\infty }\right)\\ & & +\lambda \left(S\right)\epsilon_{\infty }^{{}^{\prime }}\epsilon_{\infty }\left(2\delta_{0}+C_{ij}\delta \right), \end{array}$$

where λ(S) is the Lebesgue measure of S. This is bounded since S is compact. Let $\delta_{0}^{{}^{\prime }}>0$ be s.t. if $\max\left(||p-p_{0}{\left|\right|}_{C^{r}},||q-q_{0}{\left|\right|}_{C^{r}}\right)<\delta_{0}^{{}^{\prime }}$, then (A37) is less than $\frac{\epsilon }{4\lambda \left(S\right)\epsilon_{\infty }^{{}^{\prime }}\epsilon_{\infty }}$. Let $\delta_{1}=\frac{\epsilon }{4\lambda \left(S\right)C_{ij}\epsilon_{\infty }^{{}^{\prime }}\left(1+\epsilon_{\infty }\right)}$. Then, if $\delta <\min(\delta_{0}^{{}^{\prime }},\delta_{1})$,

$$|c\left(p,q\right)-c\left(p_{0},q_{0}\right)|<\epsilon .$$

 ☐

Given that each $c_{i}\left(p,q\right)$ is continuous, then ${\left(\sum_{i\in J}c_{i}\left(p,q\right)\right)}^{2}$ is also continuous wrt *p* and *q*.

We now argue that $\Sigma (s,K_{H})$ is compact. First, a set is relatively compact if its closure is compact. Based on the Arzela–Ascoli theorem [83], the space $\Sigma (s,K_{H})$ is relatively compact in the topology induced by the ${∥\cdot ∥}_{\Sigma (t,K_{H})}$ norm for any t<s. We choose t=r. It can then be shown that under the ${∥\cdot ∥}_{\Sigma (r,K_{H})}$ norm, $\Sigma (s,K_{H})$ is complete [82]. Since $\Sigma (s,K_{H})$ is contained in a metric space, it is also closed and therefore, equal to its closure. Thus, $\Sigma (s,K_{H})$ is compact. Then, since $\Sigma (s,K_{H},\epsilon_{0},\epsilon_{\infty })$ is closed in $\Sigma (s,K_{H})$, it is also compact. Therefore, since for each $p,q\in \Sigma (s,K_{H},\epsilon_{0},\epsilon_{\infty })$, ${\left(\sum_{i\in J}c_{i}\left(p,q\right)\right)}^{2}<\infty$, based on the Extreme Value Theorem, we have

$$\begin{array}{ccc} \underset{p,q\in \Sigma (s,K_{H},\epsilon_{0},\epsilon_{\infty })}{\sup}\mathrm{Bias}{\left({\tilde{G}}_{w_{0}}\right)}^{2} & = & \underset{p,q\in \Sigma (s,K_{H},\epsilon_{0},\epsilon_{\infty })}{\sup}\frac{\epsilon^{2}}{N}{\left(\sum_{i\in J}c_{i}\left(p,q\right)\right)}^{2}\\ & = & \frac{\epsilon^{2}}{N}C, \end{array}$$

where we use the fact that *J* is finite (see Section 3.2 or Appendix B for the set *J*).
